# Understanding Gene Expression and Transcriptome Profiling of COVID-19: An Initiative Towards the Mapping of Protective Immunity Genes Against SARS-CoV-2 Infection

**DOI:** 10.3389/fimmu.2021.724936

**Published:** 2021-12-15

**Authors:** Chiranjib Chakraborty, Ashish Ranjan Sharma, Manojit Bhattacharya, Hatem Zayed, Sang-Soo Lee

**Affiliations:** ^1^ Department of Biotechnology, School of Life Science and Biotechnology, Adamas University, Kolkata, India; ^2^ Institute for Skeletal Aging & Orthopedic Surgery, Hallym University-Chuncheon Sacred Heart Hospital, Chuncheon-si, South Korea; ^3^ Department of Zoology, Fakir Mohan University, Balasore, India; ^4^ Department of Biomedical Sciences, College of Health and Sciences, Qatar University (QU) Health, Qatar University, Doha, Qatar

**Keywords:** COVID-19, differentially expressed genes, transcriptome profiling, interactome mapping, protective immunity

## Abstract

The COVID-19 pandemic has created an urgent situation throughout the globe. Therefore, it is necessary to identify the differentially expressed genes (DEGs) in COVID-19 patients to understand disease pathogenesis and the genetic factor(s) responsible for inter-individual variability. The DEGs will help understand the disease’s potential underlying molecular mechanisms and genetic characteristics, including the regulatory genes associated with immune response elements and protective immunity. This study aimed to determine the DEGs in mild and severe COVID-19 patients versus healthy controls. The Agilent-085982 Arraystar human lncRNA V5 microarray GEO dataset (GSE164805 dataset) was used for this study. We used statistical tools to identify the DEGs. Our 15 human samples dataset was divided into three groups: mild, severe COVID-19 patients and healthy control volunteers. We compared our result with three other published gene expression studies of COVID-19 patients. Along with significant DEGs, we developed an interactome map, a protein-protein interaction (PPI) pattern, a cluster analysis of the PPI network, and pathway enrichment analysis. We also performed the same analyses with the top-ranked genes from the three other COVID-19 gene expression studies. We also identified differentially expressed lncRNA genes and constructed protein-coding DEG-lncRNA co-expression networks. We attempted to identify the regulatory genes related to immune response elements and protective immunity. We prioritized the most significant 29 protein-coding DEGs. Our analyses showed that several DEGs were involved in forming interactome maps, PPI networks, and cluster formation, similar to the results obtained using data from the protein-coding genes from other investigations. Interestingly we found six lncRNAs (TALAM1, DLEU2, and UICLM CASC18, SNHG20, and GNAS) involved in the protein-coding DEG-lncRNA network; which might be served as potential biomarkers for COVID-19 patients. We also identified three regulatory genes from our study and 44 regulatory genes from the other investigations related to immune response elements and protective immunity. We were able to map the regulatory genes associated with immune elements and identify the virogenomic responses involved in protective immunity against SARS-CoV-2 infection during COVID-19 development.

## Introduction

The COVID-19 pandemic is one of the most devastating infectious diseases in recent times, spreading rapidly to more than 188 countries. As of November 20, 2021, over 256.5 million confirmed cases were reported and nearly 5.15 million deaths (1, 2). COVID-19 has also been reported in waves globally. The second wave caused an increased number of confirmed cases and mortality in different parts of the world (3–5). COVID-19 infection can be categorized into mild and severe conditions in humans (6). In the second wave, most patients showed mild to severe symptoms. However, 15% of the COVID-19 patients progressed towards acute or severe disease, requiring hospitalization (7). Studies have been performed to understand the differences between mild infections versus severe infections in patients. In one study, viral dynamics were investigated in 76 patients whose clinical presentation was classified as mild or severe (8). In that study, 61% of patients (46 patients) were categorized as mild, and the remaining 39% of patients (30 patients) were classified as severe. Patients with mild infection cleared the virus very early, while patients with severe infection had an extended virus-shedding phase with a high viral load (8). Velavan and Meyer attempted to understand the host markers associated with mild and severe infection (9). The C-reactive protein (CRP) levels in patients with mild and severe infection were also studied to develop a predictive marker (10). Numerous other studies have also been performed to understand the molecular biological aspects, immunological impact, and pathogenicity of this infectious virus (11–13). Many efforts have been made to design and develop effective diagnostics, therapeutics, and vaccines against the virus (14–18). It is essential to understand the differences in gene expression in patients with different levels of severity of infection to help develop therapies against the virus.

To understand complex diseases, gene expression studies and network analyses are of immense importance (19, 20). It aids in understanding the underlying mechanism and genetic vulnerability to complex diseases (21). Gene expression studies can also help understand the transcriptomic landscape of cells (22). Identifying the gene regulatory networks and host immune response dynamics can help to develop therapeutics, as the transcriptomic profiling of cells during virus infection helps to understand host gene regulatory networks and the host immune response (23–25). Recently, Xiong et al. studied the transcriptomic pattern of COVID-19 patients using peripheral blood mononuclear cells (PBMCs) and various body fluids (26). Ziegler et al. determined a gene expression profile in interferon-stimulated airway epithelial cells infected by SARS-CoV-2 in humans and non-human primates (27). In another study, Jain et al. evaluated transcriptomic profiling of COVID-19 patients with mild, moderate, and severe infections (28). The differentially expressed genes were evaluated using microarray technology in patients with different severities of the disease. Microarray technology is a robust procedure that is commonly used to study differentially expressed genes to understand gene mapping, association, linkage, and expression (29, 30). However, the number of studies comparing the whole genome transcriptome of PBMCs isolated from COVID-19 patients with mild and severe infections versus healthy controls is limited.

The mapping of genes related to the activation of immune cells, immune system-related components, and protective immunity may help understand the genomic landscape and the modulator genes or proteins of any disease. It will also provide a better understanding of the immunology of the disease (31). The immune-mediated approach may aid in developing an immunotherapeutic for the treatment of COVID-19 (32, 33). In this study, we attempted to understand the expression of genes related to the activation of immune cells, immune system-related components, and protective immunity in COVID-19 patients.

This study aimed to identify the DEGs in COVID-19 patients with mild and severe symptoms versus healthy controls. With the information on significantly upregulated DEGs, we developed an interactome map, a protein-protein interaction (PPI) pattern, a cluster analysis of the PPI network, and performed pathway enrichment analysis. We also developed a transcriptome network profile, a PPI pattern, a cluster analysis of the PPI network, and a pathway enrichment analysis of top-ranked genes from three other COVID-19 gene expression studies performed by (26–28), and compared the results with those obtained from our gene expression study. Additionally, we identified the differentially expressed lncRNA genes of COVID-19 patients and constructed DEG-lncRNA co-expression networks. Finally, an attempt was made to identify the regulatory genes related to immune response elements and protective immunity combining the analyses from the three previous COVID-19 gene expression studies and this study.

## Materials and Methods

### Array Data Acquisition

**Figure 1:**
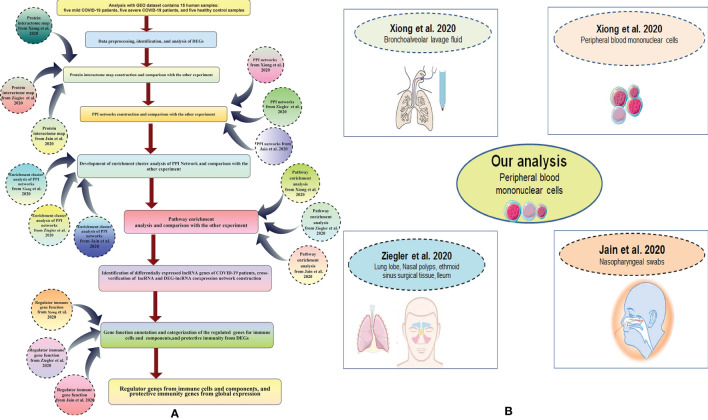
Outline of the workflow and diverse sample types of our entire study. **(A)** A brief workflow of our bioinformatics study. **(B)** Different diverse sample types of our complete study.

The GEO database, an NCBI resource, was used for data acquisition; the GSE164805 dataset was used in this study. In this dataset, gene expression was profiled through the array. GEO is the database where gene expression profiles are stored, and users can download a dataset of gene expression profiles from this database (34). We used different keywords “COVID-19”, “*Homo sapiens*”, and “Microarray” to search GEO datasets. All selected expression datasets were log-transformed expression (log2 transformed) and then standardized. The outline of gene expression and transcriptome landscape data analysis of patients with COVID-19 are shown in **Figure 1A**.

### Patients With COVID-19

All the patient data was derived from the GEO database, which is an open database from NCBI. We divided our dataset into three: COVID-19 patients with a mild infection, COVID-19 patients with severe infection, and healthy control. Our dataset contains 15 human samples: five COVID-19 patients with mild infection, five COVID-19 patients with severe infection, and five healthy control samples. Among the healthy controls, four were males, and one was female. Four males and one female were selected for the COVID-19 patient group with mild infection. All the patients with severe COVID-19 infection were males (**Table 1**).

**Table 1:** The summary of 15 human subjects (control and COVID-19 patients) datasets and study characteristics.

Group	Accession	Source name	Cell type	Disease	Gender	Age
**Control**	GSM5019817	PBMC, HC	peripheral blood mononuclear cells (PBMCs)	healthy	male	62
**Control**	GSM5019818	PBMC, HC	peripheral blood mononuclear cells (PBMCs)	healthy	male	56
**Control**	GSM5019819	PBMC, HC	peripheral blood mononuclear cells (PBMCs)	healthy	male	54
**Control**	GSM5019820	PBMC, HC	peripheral blood mononuclear cells (PBMCs)	healthy	male	71
**Control**	GSM5019821	PBMC, HC	peripheral blood mononuclear cells (PBMCs)	healthy	female	56
**Mild COVID-19** patient	GSM5019822	PBMC, mild patient	peripheral blood mononuclear cells (PBMCs)	COVID-19	male	55
**Mild COVID-19** patient	GSM5019823	PBMC, mild patient	peripheral blood mononuclear cells (PBMCs)	COVID-19	male	44
**Mild COVID-19** patient	GSM5019824	PBMC, mild patient	peripheral blood mononuclear cells (PBMCs)	COVID-19	male	51
**Mild COVID-19** patient	GSM5019825	PBMC, mild patient	peripheral blood mononuclear cells (PBMCs)	COVID-19	male	54
**Mild COVID-19** patient	GSM5019826	PBMC, mild patient	peripheral blood mononuclear cells (PBMCs)	COVID-19	female	53
**Severe COVID-19** patient	GSM5019827	PBMC, severe patient	peripheral blood mononuclear cells (PBMCs)	COVID-19	male	54
**Severe COVID-19** patient	GSM5019828	PBMC, severe patient	peripheral blood mononuclear cells (PBMCs)	COVID-19	male	52
**Severe COVID-19** patient	GSM5019829	PBMC, severe patient	peripheral blood mononuclear cells (PBMCs)	COVID-19	male	73
**Severe COVID-19** patient	GSM5019830	PBMC, severe patient	peripheral blood mononuclear cells (PBMCs)	COVID-19	male	51
**Severe COVID-19** patient	GSM5019831	PBMC, severe patient	peripheral blood mononuclear cells (PBMCs)	COVID-19	male	60

### Data Preprocessing, Identification, and Analysis of DEGs

To analyze raw gene expression data, we used the statistical tool GEO2R, and this tool further uses the R/Bioconductor and limma package (34, 35). We developed different types of statistical plots using RStudio. The statistical plots are volcano plots, mean difference (MD) plots, uniform manifold approximation and projection (UMAP) plot, venn diagram, box plot, expression density plot, adjusted p-value histogram, moderated t-statistic quantile-quantile (q-q) plot, and mean-variance trend plot. These plots were used to identify and analyze DEGs using PBMCs from the different groups: COVID-19 patients with a mild infection, COVID-19 patients with severe infection, and healthy controls (36–38). The dataset’s principal standards were set to | log (fold change) | > 1 and p < 0.05 to analyze and acquire significant DEGs.

### Acquisition of Gene Expression Data From Other Studies

We acquired the top-ranking genes from other studies to compare gene expression and transcriptome profiling. We generated top-ranking genes from various studies conducted by (26–28). Xiong et al. performed a gene expression study using bronchoalveolar lavage fluid and peripheral blood mononuclear cell samples. The researchers used RNA sequencing library construct for RNA library construction and high-throughput RNA sequencing for gene expression studies (26). Ziegler et al. performed a gene expression study using lung lobe, nasal polyps, ethmoid sinus surgical tissue, and ileum samples; they used a single-cell RNA-sequencing assay for gene expression studies (27). Jain et al. analyzed gene expression profiles using nasopharyngeal swab samples; they used shotgun transcriptome sequencing of RNA for their gene expression profiling study (28). The acquired top-ranking expressed genes of COVID-19 patients from the different studies are shown in **Table 2**. Our study mapped gene expression from the diverse sample types (**Figure 1B**).

**Table 2:** List of top ranking expressed genes of COVID-19 patients from the different experiments.

Sl. No	Group name	Sample type	Assay	Gene expression	Reference
1.	Xiong et al., 2020	Bronchoalveolar lavage fluid	RNA library construction, high-throughput RNA sequencing	CXCL1, CXCL2, CXCL6, CXCL8, IL 33, CXCL10/IP-10, CCL2/MCP-1,CCL3/MIP-1A, CCL4/MIP1B	(26)
2.	Xiong et al., 2020	Peripheral blood mononuclear cells	RNA library construction, high- throughput RNA sequencing	CXCL10, TNFSF10, TIMP1, C5, IL18, AREG, NRG1, IL10, ADA2, HK1, GAT1, PGD, PLA2G15, CTSD, GAA, LAIR1	(26)
3.	Ziegler et al., 2020	Lung lobe, Nasal polyps, ethmoid sinus surgical tissue, Ileum	Single-cell RNA-sequencing	IFNGR2, TRIM27, NT5DC1, ARL6IP1, IFNAR1, TMPRSS2, ACE2, TRIM28, APOA1, FABP6, ENPEP, STAT1, IFI6, IFITM1, GBP2, FI35, XAF1	(27)
4.	Jain et al., 2020	Nasopharyngeal swabs	Shotgun transcriptome sequencing of RNA	CXCL5, CXCL12, CCL2, CCL4, CXCL10, IFIH1, IFI44, IFIT1, IL6, IL10, CSF2, TNFSF11, TNFRSF11B, IL18R1, BMP2, BMP7, PDGFA, IFIT1B, C4BPA, CCR6, CCR22, CCR25, IL3RA, IL11, IL19, IL21RA, SERPINE1, SERPINF2	(28)

### The Protein Interactome—Construction and Comparison With Other Studies

To understand the associations between the DEGs from our dataset, we constructed a protein interactome using HuRI (39). A protein interactome was generated through binary protein interactions using approximately 53,000 high-quality PPIs. We also developed a transcriptome network by acquiring data from the other three studies (26–28).

### Development of PPI Networks and Comparison With Other Experiments

To understand the associations between protein-coding DEGs, we constructed a PPI network using the web-based tool STRING (40, 41). The cut-off criteria were fixed with a confidence interaction score ≥ of 0.4 to obtain consistency from the dataset for the PPI interactions. The PPI network analysis outcome was represented by Cytoscape from STRING to better understand and conceptualize the PPI interactions among the highly DEGs (42, 43). STRING can integrate data from several resources: ConsensusPathDB, HitPredict, IMP, IMID, VisANT, GeneMANIA, and I2D.

Simultaneously, we have generated a PPI network that acquired top-ranking expressed genes of COVID-19 patients from the other studies (26–28). We compared all PPI networks.

### Development of Enrichment Cluster Analysis of PPI Network and Comparison With Other Studies

For cluster analysis, we generated similarities between intra-cluster and inter-cluster. We transformed the outcomes from Metascape (44) using the Cytoscape software.

Metascape performed cluster analysis experiments using different databases such as InWeb_IM (45), BioGrid (46), and OmniPath (45), and the MCODE algorithm. In this study, the relationships’ capture condition is a subset of enriched terms selected and rendered as a network plot. In this case, the tool has a condition with a similarity of > 0.3 connected by edges. The terms of selection were set with the best p-values from each of the 20 clusters.

We also developed different clusters of PPI networks using top-ranking expressed genes of COVID-19 patients from the studies (26–28). We compared all the cluster analyses of the PPI network.

### Functional Pathway Enrichment Analysis and Comparison With Other Studies

Pathway enrichment analysis was performed using Metascape analysis (45) with the 29 significantly expressed genes that use different ontology sources: GO biological processes, KEGG pathway, Reactome gene sets, and so on. The study used a term with a p-value < 0.01, with a minimum count of 3. The q-values were computed using a significant process, which accounts for multiple tests. This process is called the Benjamini-Hochberg procedure (47). Similar to the previous analysis, we performed pathway enrichment analysis using top-ranking expressed genes of COVID-19 patients from the different studies (26–28). We compared all the results of the pathway enrichment analysis.

### Identification of Differentially Expressed LncRNA Genes in COVID-19 Patients and Cross-Verification of the Construction of the LncRNA and the DEG-LncRNA Co-Expression Networks

We mapped the top-ranking differentially expressed lncRNA genes from the 250 DEGs. Using top-ranking differentially expressed lncRNA genes and other DEGs, we constructed DEG-lncRNA pairs networking using the Cytoscape software (44). In this case, the Cytoscape MCODE plug-in was used (48). Before network construction, we cross-verified the lncRNA through a non-coding RNA sequence database, RNAcentral (49), a database for subcellular localization of lncRNAs.

### Gene Function Annotation and Categorization of Regulated Genes Related to Immune Response Elements And Protective Immunity From DEGs

We attempted to map the genes from the DEGs with immunomodulatory and protective immunity properties. We used NCBI Genbank (50) and GeneCards (51, 52).

## Results

### Data Acquisition of Patients With COVID-19 and DEG Profiling

**Figure 2:**
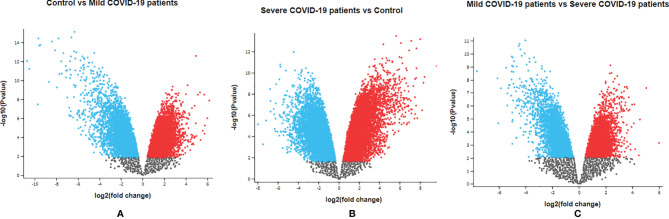
Visualization of identified DEGs using volcano plots**. (A)** DEG volcano plot using the data of control vs. mild COVID-19 patients. **(B)** DEG volcano plot using the data of severe COVID-19 patients vs. control healthy volunteers. **(C)** DEG volcano plot using the data of mild COVID-19 patients vs. severe COVID-19 patients. In this figure, red dots denote upregulated DEGs and blue dots denote downregulated DEGs.

Our study analyzed the gene expression profiles and transcriptome landscape of the GSE164805 dataset from the GEO database. The Agilent-085982 Arraystar human lncRNA V5 microarray platform was used for this expression analysis. In this study, PBMCs were taken from COVID-19 patients with mild and severe infections and healthy controls for gene expression analysis. Our dataset containing 15 human samples in three groups (the two COVID-19 patients groups and one healthy control group) (**Table 1**). The volcano plot is a statistical plot, and it is a type of scatter plot that shows p-value (statistical significance) against fold change (magnitude of change). The top 250 DEGs were ranked in this study (**Tables S1**–**S3**). **Table S1** describes the ID, p-value, F, and gene description of the top 250 DEGs. **Table S2** describes the top 250 DEG sequences, and **Table S3** represents the accession number and chromosome of the top 250 DEGs. The developed volcano plot of DEGs, and the significant genes showed the satisfactory value which was created using the dataset (**Figure 2**). Using the cut-off criteria (p < 0.05 and |log2 FC|>1), the upregulated DEGs were acquired. We next developed the DEG volcano plot using the data of control vs. COVID-19 patients with mild infection (**Figure 2A**). Similarly, we also developed another DEG volcano plot using the data of COVID-19 patients with severe infection vs. control healthy volunteers (**Figure 2B**). At the same time, we illustrated the DEG volcano plot comparing the data of COVID-19 patients with mild vs. severe infections (**Figure 2C**). In all cases, DEGs of volcano plots were adjusted with a p-value cut-off of 0.05. Red dots represent the upregulated DEGs, and the blue dots represent the downregulated DEGs.

**Figure 3:**
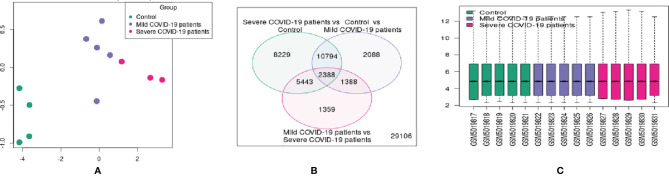
Visualization of identified DEGs using MD**. (A)** DEGMD plot using the data of control vs. mild COVID-19 patients. **(B)** DEGMD plot using the data of severe COVID-19 patients vs. control healthy volunteers. **(C)** DEGMD plot using the data of mild COVID-19 patients vs. severe COVID-19 patients. In this figure, red dots denote upregulated DEGs, and blue dots denote downregulated DEGs.

For the visualization of the DEGs, we also developed an MD plot. The plot helps to demonstrate the log2 fold change against average log2 expression values, and here, the adjusted p-value cut-off was 0.05. This study also depicted an MD plot to understand the log2 fold change against average log2 expression values (**Figure 3**). **Figure 3A** shows the DEG MD plot using the data of control vs. COVID-19 patients with mild infection. **Figure 3B** depicts the DEG MD plot using COVID-19 patients with severe infection vs. control healthy volunteers. **Figure 3C** depicts the DEG MD plot using the data of COVID-19 patients with mild vs. severe infections. In all cases, red dots represent the upregulated DEGs, and blue dots represent the downregulated DEGs.

**Figure 4:**
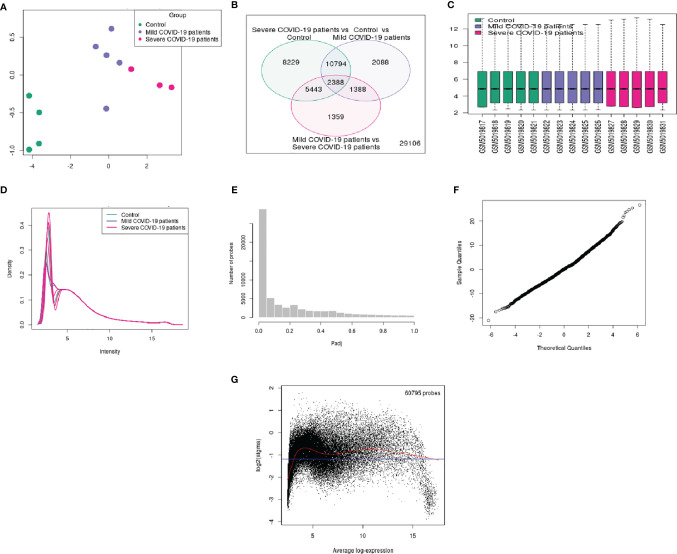
Different types of statistical plots were developed from our study. **(A)** UMAP plot visualizing samples related to each other. **(B)** Venn diagram shows the groups’ common genes. **(C)** The box plot shows the distribution of the selected samples’ values for this study. **(D)** The expression density plot shows the distribution of values of the DEGs of the three groups. **(E)** The adjusted p-value histogram represents the p-value in the experiment (the top DEGs). **(F)** The moderated t-statistic q-q plot shows our DEGs data sample against the theoretical quantiles a Student’s t distribution. **(G)** The mean-variance trend plot shows the mean-variance relationship of the gene expression data.

For better visualization, we represented the DEG data using several other statistical plots. First, we have developed an UMAP plot (**Figure 4A**). It is a dimension reduction procedure useful for visualizing samples that are related to each other. Our analysis detected the control, mild, and severe samples. We depicted one Venn diagram, which shows the common DEGs among the three groups the “COVID-19 patient with severe infection vs. control healthy volunteers” groups and the “control vs. COVID-19 patients with mild infection” groups show the 10794 DEGs those significant genes that are common to both contrasts (**Figure 4B**).

Similarly, 2338 DEGs were typical for both of our groups. Therefore, we depicted a box plot from the dataset, which informs us of the distribution of the selected samples’ values for this study (**Figure 4C**). The data distribution indicated that the data could be useful and suitable for the DEGs analysis. We also analyzed our dataset and developed an expression density plot of the distribution of values of the DEGs of the three groups (**Figure 4D**). Another adjusted p-value histogram that was developed showed that the p-value in the experiment is identical to that of the top DEGs. In this histogram, the p-values are relatively consistent (**Figure 4E**). We also illustrated the moderated t-statistic q-q plot quantiles of our DEGs’ data sample against the theoretical quantiles of a Student’s t-distribution (**Figure 4F**). In our study, values recline along a straight line, which indicates that the investigation and data of the DEGs are ideal. Therefore, the values for the DEGs of our sample quantiles follow the distribution of theoretical quantiles.

**Figure 5:**
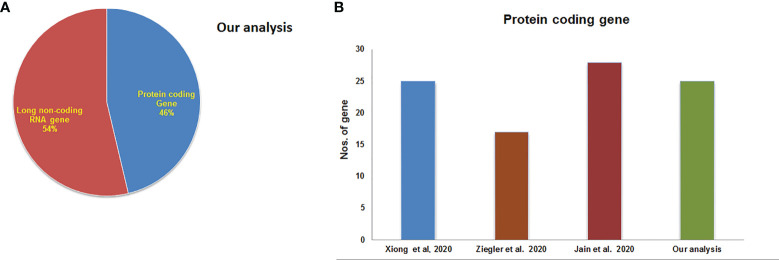
Significantly expressed genes from our experiments and other experiments. **(A)** The percentage of significantly expressed protein-coding genes and lncRNAs. **(B)** Total no. of top-ranking protein-coding genes from our study and other studies.

Finally, the mean-variance trend plot, which shows the mean-variance relationship of the gene expression data has been shown (**Figure 4G**). Each dot represents a gene, and the statistical plot is described after fitting a linear model. The average log expression line shows that the values of the early DEGs are highly dense. We have listed the significant DEGs (both protein-coding genes and long non-coding RNAs) from our studies in **Table 3**. From the significant DEGs, we found several protein-coding genes and lncRNA. The percentage of significantly expressed protein-coding genes and lncRNAs is depicted through a pie diagram (**Figure 5A**). **Table S4** describes all protein-coding genes from the 250 DEGs and their NCBI accession numbers and gene names.

**Table 3:** Significantly upregulated protein-coding genes DEGs from three experimental human groups of our dataset.

Sl. No.	Gene name	P-value	F-value
1.	CERKL	1.95e-10	135
2.	EIF4G1/EIF3G/EIF3E	1.94e-09	97.9
3.	RPL18A	2.14e-10	133.3
4.	EXOSC2/EXOCS5	2.86e-09	92.7
5.	STRN4	5.94e-10	115.7
6.	RPL3L/RPL35/RPL1BA/RPL19	1.37e-10	141.8
7.	RPS3/RPS16	2.14e-10	133.3
8.	SMTN	2.03e-09	97.3
9.	FGF1	3.73e-10	123.4
10.	PPP1R12A	2.63e-09	93.8
11.	CNNM2	9.07e-10	109
12.	AP2M1	1.68e-09	100
13.	EDN1	4.12e-10	121.7
14.	ARHGEF1	2.21e-12	248.8
15.	DUS1L	1.49e-09	101.7
16.	RBM5	3.73e-10	123.4
17.	MPHOSPH6	2.99e-09	92.2
18.	SKIV2L2	2.86e-09	92.7

### Gene Expression Data of the Different COVID-19 Patients From the Other Studies

The acquired top-ranking expressed genes of COVID-19 patients from the various studies are shown in **Table 2**. The number of protein-coding genes in our investigation is represented through a bar diagram in **Figure 5B**. This figure also shows the total numbers of top-ranking protein-coding genes from the other studies.

### Construction of the Protein Interactome Map and Comparison With Other Studies

**Figure 6:**
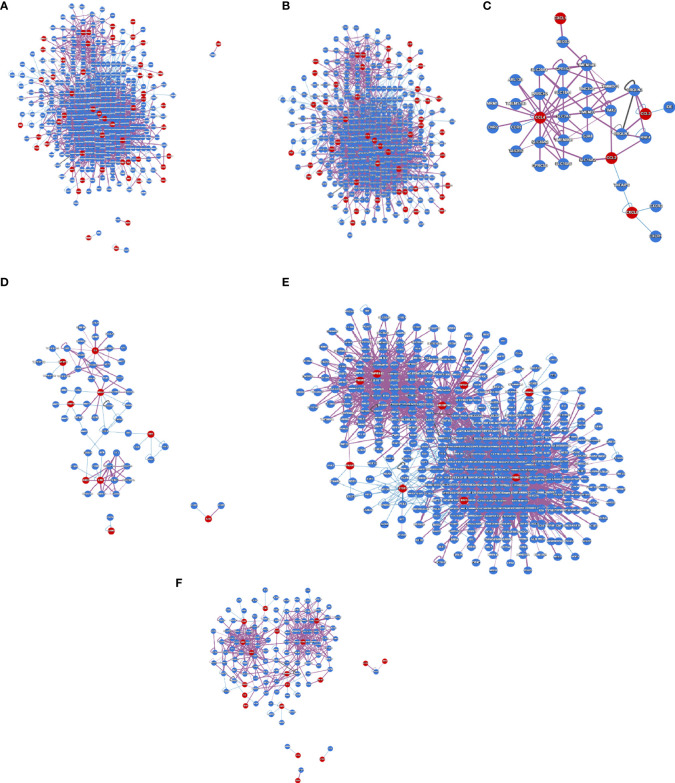
The protein interactome map constructed using protein-coding DEGs from our study and other studies. **(A)** The protein interactome map with protein-coding genes from the 250 DEGs in our research. **(B)** The protein interactome map of the central cluster from our study was identified from **(A)**. **(C)** The protein interactome map from Xiong et al. study where the samples were the bronchoalveolar lavage fluid. **(D)** The protein interactome map with the top-ranked genes of the Xiong et al. study from the PBMC samples. **(E)** The protein interactome map with data from the Ziegler et al. study where the samples were collected from the lung lobe, nasal polyps, ethmoid sinus surgical tissue, and ileum. **(F)** The protein interactome map with the top-ranked genes of the Jain et al. study that used nasopharyngeal swab sample.

Protein interactions within a cell can be represented through a protein interactome map, providing global insights into genome function and cellular organization. It will provide a comprehensive understanding of the interactome networks of SARS-CoV-2-infected human cells. We developed a protein interactome with protein-coding genes from the 250 DEGs in our study (**Figure 6A**). We found that the number of interactions was 2901 and the number of proteins that participated in the interactions was 453, and the average node degree was 12.53. Next, we depicted the protein interactome of the central cluster from our previous study (**Figure 6B**). We also portrayed the protein interactome using the data from the Xiong et al. study where the samples were the bronchoalveolar lavage fluid (**Figure 6C**). We found that the number of interactions was 76, the number of proteins that participated in the interactions was 35, and the average node degree was 4.06. We then illustrated the protein interactome with the top-ranked genes of the Xiong et al. study from the PBMC samples (**Figure 6C**). Here, we found that the number of interactions was 103, the number of proteins that participated in the interactions was 62, and the average node degree was 3.11. Similarly, we represented one protein interactome with data from the Ziegler et al. study where the samples were collected from the lung lobe, nasal polyps, ethmoid sinus surgical tissue, and ileum (**Figure 6E**). We found that the number of interactions was 2613, the number of proteins that participated in the interaction was 504, and the average node degree was 10.15. Finally, we have illustrated the protein interactome with the top-ranked genes of the Jain et al. study that used nasopharyngeal swabs as samples (**Figure 6F**). We found that the number of interactions was 437, the number of proteins that participated in the interaction was 128, and the average node degree was 6.56.

### PPI Network Analysis and Comparison With Other Studies

**Figure 7:**
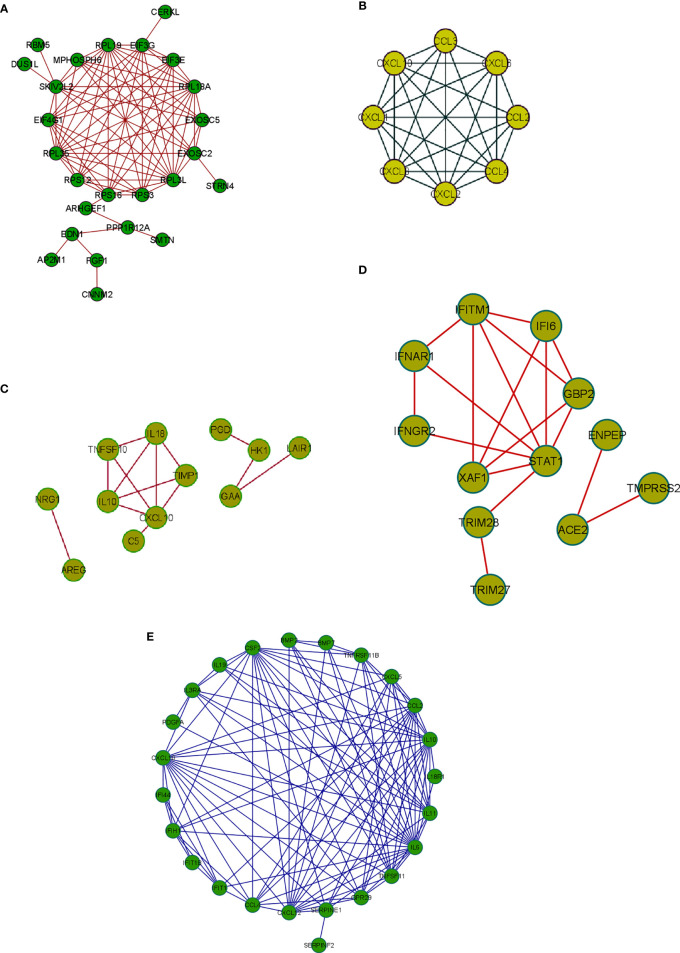
The PPI network constructed using protein-coding DEGs from our study and other studies. **(A)** The PPI network with significant protein-coding DEGs in our research. **(B)** The PPI network from Xiong et al. study where the samples were the bronchoalveolar lavage fluid. **(C)** The PPI network with the top-ranked genes of the Xiong et al. study from the PBMC samples. **(D)** The PPI network with data from the Ziegler et al. study and the gene expression data was collected from the lung lobe, nasal polyps, ethmoid sinus surgical tissue, and ileum. **(E)** The PPI network with the top-ranked protein-coding genes used a nasopharyngeal swab sample (the Jain et al. study).

Integrative gene expression analysis and creating the PPI networks from the DEGs coding proteins are essential to understanding the diseases’ molecular pathology. The PPI network analysis also showed the functional and physical associations among DEGs’ coding proteins of other samples of COVID-19. From this analysis we depicted a PPI using significant protein-coding genes from the 250 DEGs in our study (**Figure 7A**). At the same time, we have also developed a PPI using the top-ranked genes of the Xiong et al. study that used bronchoalveolar lavage fluid as samples (**Figure 7B**). Similarly, we depicted a PPI using the top-ranked genes of the Xiong et al. study using the PBMCs as samples (**Figure 7C**). Again our study illustrates a PPI using the top-ranked genes from the Ziegler et al. study (**Figure 7D**). Finally, we depicted a PPI using the top-ranked genes of the Jain et al. study (**Figure 7E**).

### Enrichment Cluster Analysis of the PPI Network and Comparison With Other Studies

**Figure 8:**
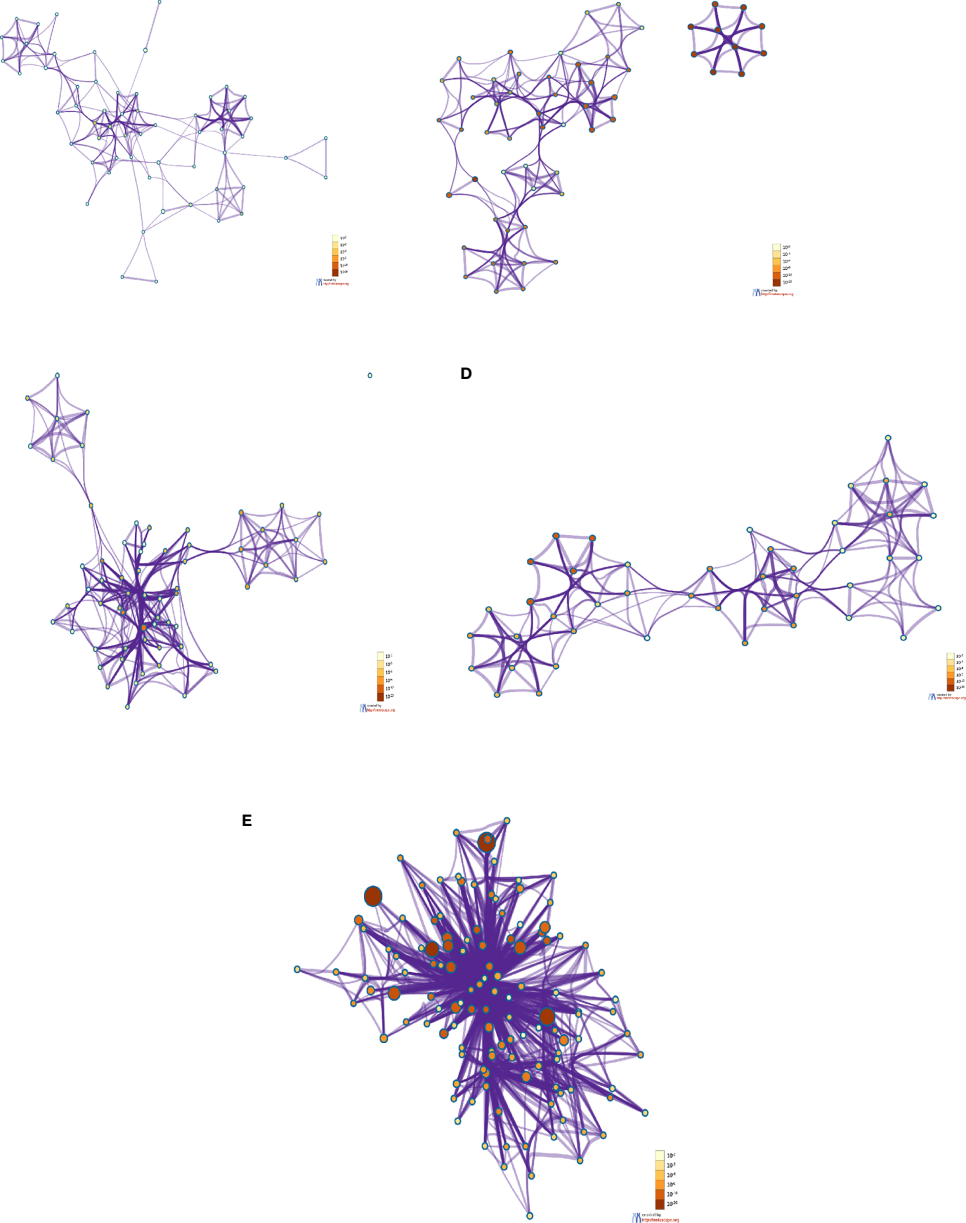
The enrichment cluster analysis of the PPI network using protein-coding DEGs from our study and other studies. **(A)** The enrichment cluster analysis of the PPI network with significant protein-coding DEGs in our research. **(B)** The enrichment cluster analysis of the PPI network from Xiong et al. study where the samples were the bronchoalveolar lavage fluid. **(C)** The enrichment cluster analysis of the PPI network with the Xiong et al. study’s top-ranked genes from the PBMC samples. **(D)** The enrichment cluster analysis of the PPI network with data from the Ziegler et al. study where the samples were collected from the lung lobe, nasal polyps, ethmoid sinus surgical tissue, and ileum. **(E)** The enrichment cluster analysis of the PPI network with the top-ranked protein-coding genes of the Jain et al. study that used nasopharyngeal swab sample.

The enrichment network cluster shows the intra-cluster and inter-cluster similarities from the input genes involved in different biological processes, enzymatic functions, and protein localization. It shows similarities of the other cluster proteins from the DEGs as per their function. In this study, we have developed a PPI network enrichment cluster using significant protein-coding genes from the 250 DEGs (**Figure 8A**). At the same time, our analysis represents the enrichment cluster of the PPI network using the top-ranked genes of the Xiong et al. study where bronchoalveolar lavage fluid was used as samples (**Figure 8B**). Again, we depicted the enrichment cluster of the PPI network using data from the Xiong et al. study that used the PBMC as samples (**Figure 8C**). Similarly, we developed a PPI network enrichment cluster using data from the Ziegler et al. study (**Figure 8D**). Finally, the analysis depicted the enrichment cluster of the PPI networks using data from the Jain et al. study (**Figure 8E**).

### Functional Pathway Enrichment Analysis and Comparison With Other Studies

This analysis helps researchers provide mechanistic insights into the DEGs (gene list) generated from genome-scale (omics) experiments. In this pathway enrichment analysis, gene list enrichment was identified in the COVID-19 categories and transcription factor targets.

**Figure 9:**
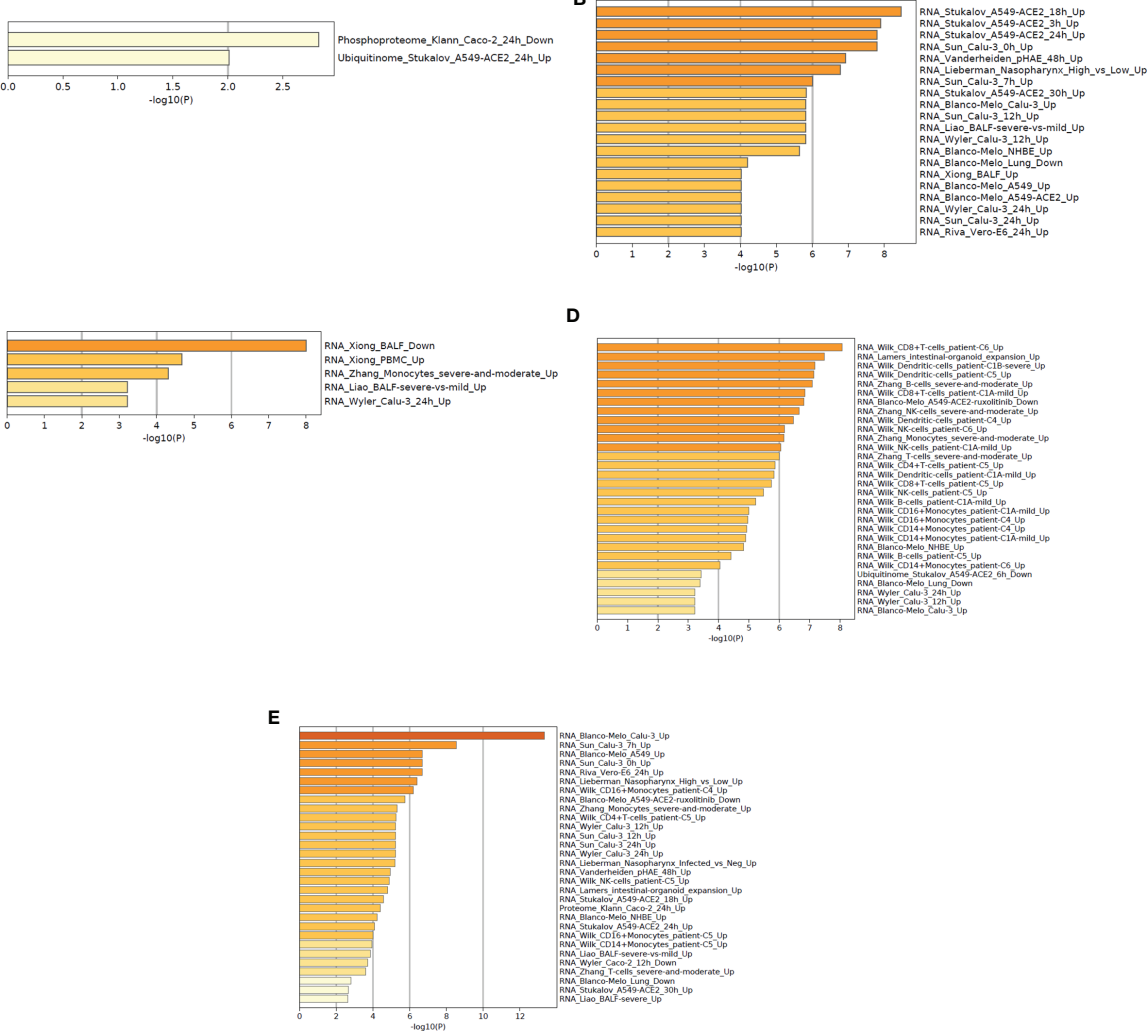
The functional pathway enrichment analysis in COVID categories using protein-coding DEGs from our study and other studies. **(A)** Functional pathway enrichment analysis in COVID-19 categories from the 250 DEGs of our study. **(B)** Functional pathway enrichment analysis in COVID-19 categories with the top-ranked genes of the Xiong et al. study that used bronchoalveolar lavage fluid sample. **(C)** Functional pathway enrichment analysis in COVID-19 categories with the top-ranked genes of the Xiong et al. study that used PBMC samples. **(D)** Functional pathway enrichment analysis in COVID-19 types with the Ziegler et al. study. **(E)** Functional pathway enrichment analysis in COVID-19 categories with the Jain et al. study.

**Figure 10:**
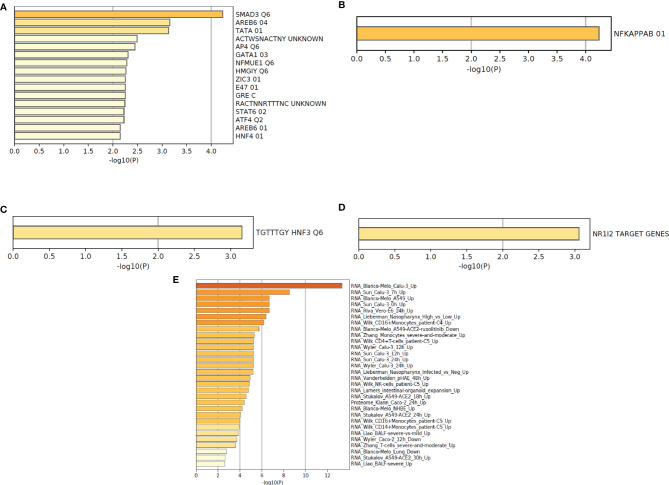
The functional pathway enrichment analysis in transcription factor targets using protein-coding DEGs from our study and other studies. **(A)** Functional pathway enrichment analysis in transcription factor targets from the 250 DEGs of our study. **(B)** Functional pathway enrichment analysis in transcription factor targets with the top-ranked genes of the Xiong et al. study that used bronchoalveolar lavage fluid sample. **(C)** Functional pathway enrichment analysis in transcription factor targets with the top-ranked genes of the Xiong et al. study that used PBMC samples. **(D)** Functional pathway enrichment analysis in transcription factor targets with the top-ranked genes of the Ziegler et al. study. **(E)** Functional pathway enrichment analysis in transcription factor targets with the top-ranked genes of the Jain et al. study.

At first, we have depicted the gene list enrichments in COVID-19 categories from the 250 DEGs of our study (**Figure 9A**). Subsequently, we developed the gene list enrichments in COVID-19 categories with the top-ranked genes of the Xiong et al. study that used bronchoalveolar lavage fluid as samples (**Figure 9B**). Similarly, we illustrated the gene list enrichments in COVID-19 categories with the top-ranked genes of the Xiong et al. study that used PBMC samples (**Figure 9C**). Then, we have developed the gene list enrichments in COVID-19 categories with the top-ranked genes of the Ziegler et al. study (**Figure 9D**). Finally, we have developed the gene list enrichments in COVID-19 categories with the top-ranked genes of the Jain et al. study (**Figure 9E**). We have developed the gene list enrichment in transcription factor targets from the 250 DEGs of our study (**Figure 10A**). Our analysis illustrates the gene list enrichment in transcription factor targets of the top-ranked genes of the Xiong et al. study that used bronchoalveolar lavage fluid as samples (**Figure 10B**). Similarly, we have depicted the identified gene list enrichment in transcription factor targets of the top-ranked genes of the Xiong et al. study where the samples were PBMCs (**Figure 10C**). At the same time, our analysis represents the gene list enrichment in transcription factor targets of the top-ranked genes of Ziegler et al. study (**Figure 10D**). At last, this analysis depicted the gene list enrichment in transcription factor targets of the top-ranked genes of the Jain et al. study (**Figure 10E**).

### Identification of Differentially Expressed LncRNA Genes In COVID-19 Patients and Cross-Verification of the Construction of the LncRNA and DEG-lncRNA Co-Expression Networks

**Figure 11:**
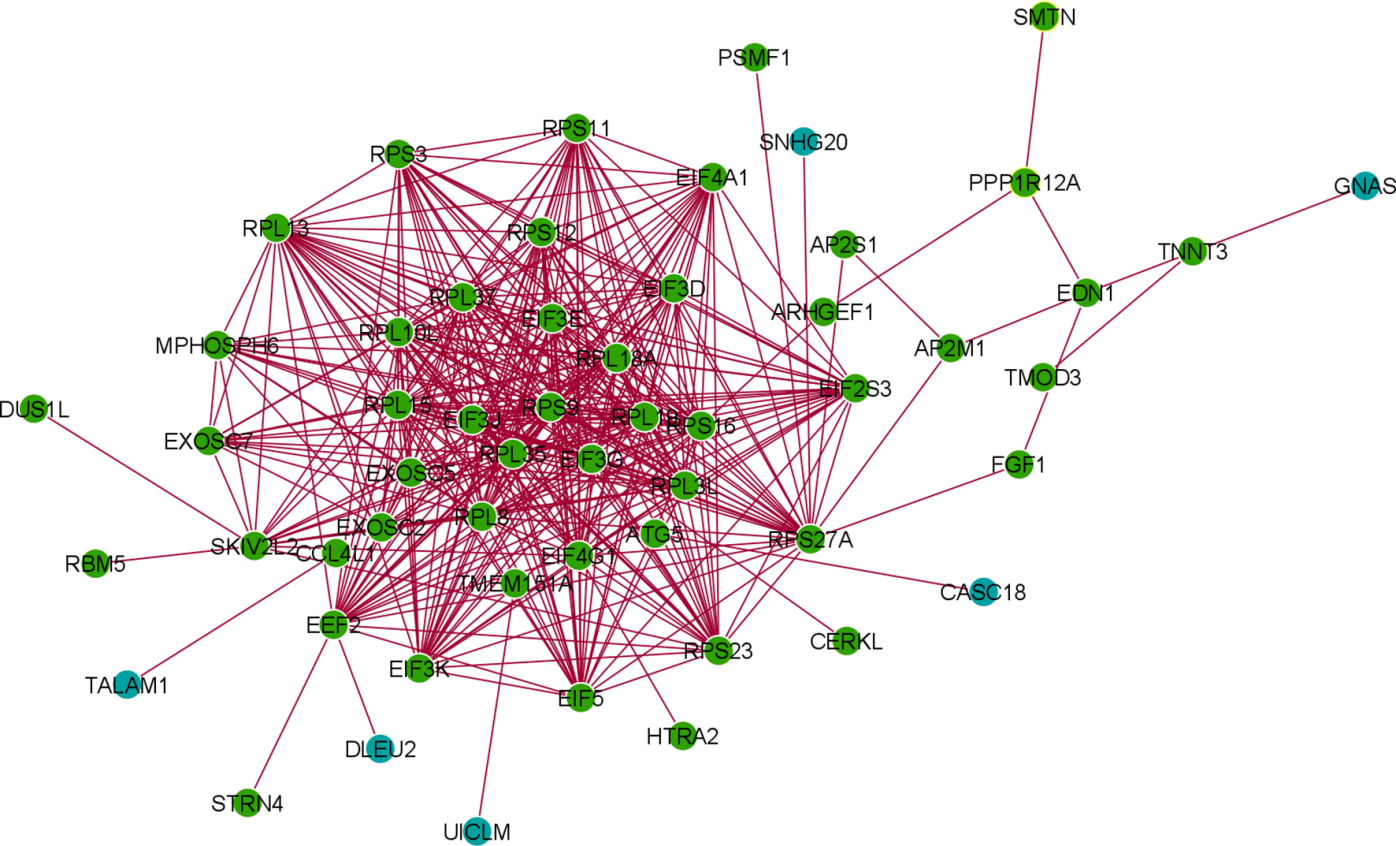
The co-expression networks of the protein expression genes of DEG and lncRNA.

**Table S5** describes differentially expressed lncRNA genes from the 250 DEGs and their NCBI accession number and gene name. **Table 4** represents the significantly upregulated DEGs lncRNA genes from the three patients groups in our dataset, and GEO2R was used to identify it. Furthermore, we cross-verified lncRNAs and identified 24 significant lncRNA genes, which are recorded in **Table 4**. We developed the co-expression networks of the protein expression genes of DEG and lncRNA (**Figure 11**).

**Table 4:** Significantly upregulated lncRNA genes from DEGs between three experimental human groups of our dataset.

Sl. No.	Gene name	P- value	F -value
1.	LOC101929613	2.60e-15	612.8
2.	LOC105370401	6.88e-15	538.5
3.	TMEM252-DT	2.91e-11	175.4
4.	HOXC13-AS	3.12e-11	173.7
5.	SEMA3B-AS	4.59e-11	164.8
6.	MIR210HG	5.25e-11	161.8
7.	DLEU2	5.71e-11	159.9
8.	MEF2C-AS2	6.53e-11	157
9.	LINC01639	3.58e-10	124.1
10.	SNHG20	6.75e-10	113.6
11.	SLC25A48-AS1	3.86e-10	122.8
12.	TALAM1	3.88e-10	122.8
13.	LOC105370619	4.66e-10	119.6
14.	UICLM	4.96e-10	118.6
15.	NRSN2-AS1	5.49e-10	116.9
16.	GNAS	7.29e-10	112.4
17.	LINC02612	1.32e-09	103.5
18.	LINC02582	1.46e-09	102
19.	LINC02000	1.47e-09	101.9
20.	LINC01393	1.48e-09	101.7
21.	LINC01191	2.19e-09	96.3
22.	N4BP2L2	2.71e-09	93.4
23.	LINC01920	2.78e-09	93.1
24.	CASC18	3.14e-09	91.5

### Gene Function Annotation and Categorization of Regulated Genes Related to Immune Response Elements and the Protective Immunity From the DEGs

**Figure 12:**
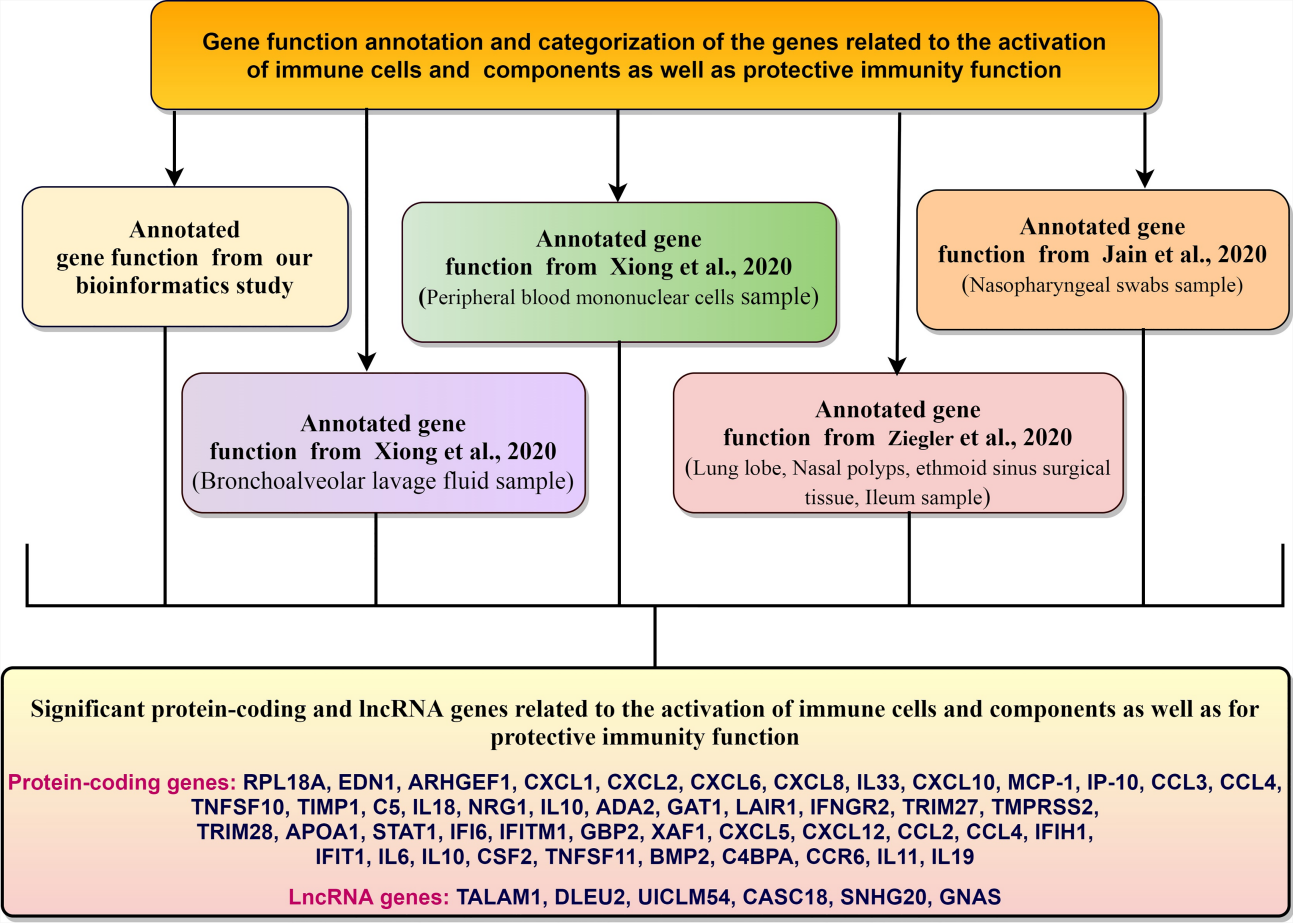
The genes associated with the immune response elements and protective immunity from DEGs.

Our gene function annotation and categorization show the genes associated with the immune response elements and protective immunity from DEGs (**Figure 12**). The genes we identified for the activation of immune cells and components, and protective immunity correlate with the other networks (**Table 5**).

**Table 5:** Annotated genes related to the activation of immune cells and components as well as for protective immunity function from our analysis and other experiments.

Sl. No.	Gene name	Remark	Ref.
***Protein coding gene (our bioinformatics study)***			
19.	RPL18A	Activation role of T cell proliferation	(53)
20.	EDN1	Involve in TLR4 responses	(54)
21.	ARHGEF1	Antigen-specific antibody production/humoral immune response	(55)
***Protein coding gene [study from (*** 26 ***)]***			
1.	CXCL1	Regulation of IL-1β level within tissue	(56)
2.	CXCL2	Self-regulated neutrophil recruitment and function	(57)
3.	CXCL6	Act as potent pro-inflammatory neutrophil chemoattractant and activator component	(58)
4.	CXCL8	Synthesis of IL-8 and important role in systemic inflammatory response syndrome	(59)
5.	IL33	Synthesis of intracellular IL-33 may play role in pro-inflammatory signaling	(60)
6.	CXCL10	Regulator of the interferon response, specially attracts activated T lymphocytes	(61)
7.	MCP-1	Activation and migration of leukocytes	(62)
8.	IP-10	Secretion of cytokines	(63)
9.	CCL3	Induction of antigen-specific T cell responses	(64)
10.	CCL4	Activation of antigen-presenting cells and B cells	(65)
11.	TNFSF10	Role in adaptive immune system	(66)
12.	TIMP1	Stimulates the immune response in lung cells	(67)
13.	C5	Protease function as membrane attack complex (MAC)	(68)
14.	IL18	Regulating the T helper responses and stimulating interferon gamma production	(69)
15.	NRG1	Regulatory role in neuroinflammation	(70)
16.	IL10	Enhance the B cell survival, proliferation, and antibody production	(71)
17.	ADA2	Regulation of immune cells (neutrophils, monocytes, NK cells and B cells) activation and survival	(72)
18.	GAT1	Decreases T cell proliferation	(73)
19.	LAIR1	Regulates the inhibition of NK cell–mediated cytotoxicity	(74)
***Protein coding gene [study from (*** 27 ***)]***			
1.	IFNGR2	Regulation of NK cell activity and B cell function	(75)
2.	TRIM27	Lowering the function of IFN and pathogen-recognition receptors	(76)
3.	TMPRSS2	Neutralizing antibodies by protease activity	(77)
4.	TRIM28	Regulation of IFN-β, IFN-γ and cytokine expression in infected lung cells	(78)
5.	APOA1	Involved in inflammatory and immune response regulation	(79)
6.	STAT1	Maturation, stability of cytotoxic and helper T cells	(80)
7.	IFI6	It delays type I interferon-induced apoptosis in cells	(81)
8.	IFITM1	Regulate the CD4+ T helper cell differentiation	(82)
9.	GBP2	Innate immune functions against intracellular pathogens	(83)
10.	XAF1	Helps in IFN-β-induced apoptosis	(83)
***Protein coding gene [study from (*** 28 ***)]***			
25.	CXCL5	Encodes receptor protein to recruit neutrophils	(84)
26.	CXCL12	Coded protein paly role in immune surveillance, inflammation response.	(85)
27.	CCL2	Activation and migration of leukocytes	(86)
28.	CCL4	Activation of antigen-presenting cells and B cells	(65)
29.	IFIH1	Involved in immune response and antiviral activity	(87)
30.	IFIT1	Encoded protein may inhibit viral replication and translational initiation	(88)
31.	IL6	Encoded cytokines functions in inflammation and the maturation of B cells	(89)
32.	IL10	It lowering the expression of Th1 cytokines, MHC class II Ags, and costimulatory effects on macrophages	(90)
33.	CSF2	It controls the production, differentiation, and function of granulocytes and macrophages	(91)
34.	TNFSF11	Regulation of T cell dependent immune response	(92)
35.	BMP2	It regulate thymic T cell development, maintain TR cell	(93)
36.	C4BPA	It controls the activation of the complement cascade	(94)
37.	CCR6	Regulate the migration and recruitment of dendritic and T cells	(95)
38.	IL11	Stimulate the T-cell-dependent development of immunoglobulin producing B cells	(96)
39.	IL19	Encoded cytokine induces the expression of IL6 and TNF-alpha and helps in inflammatory responses	(97)

## Discussion

### Main Findings of the Study

High-throughput technologies such as DNA microarrays and next-generation sequencing are beneficial for discoveries in the biomedical field. Gene expression profiling using microarray is a promising way to gain insight into the intrinsic molecular pathways, which helps to understand the complex machinery of biological systems (98, 99). We used these gene expression profiling methods to identify genes that are differently expressed in the PBMCs of COVID-19 patients with mild or severe infections compared with healthy volunteers. Zhang and Diao submitted the dataset in the GEO database. They have illustrated the antiviral and inflammation mechanisms related to the immune response associated with severe COVID-19 patients (100). However, using their dataset, we have analyzed the dataset differently. Our study investigated the DEGs in PBMCS of five patients having mild COVID-19, five patients with severe COVID-19, and five healthy volunteers using the GSE164805 dataset. We opine that our findings will help better understand the pathogenesis of SARS-CoV-2 and the host gene response during infection.

Here we have performed a comprehensive analysis of the DEGs and comparison of three groups of human subjects using advanced methods and statistical techniques. We extracted the 250 top-ranked DEGs using GEO2R and further evaluated them. Our study listed the significant DEGs (both protein-coding genes and long non-coding RNAs), and some of the significant DEGs, CERKL, RPL18A, STRN4, RPL3L/RPL35/RPL1BA/RPL19, RPS3/RPS16, AP2M1, EDN1, ARHGEF1, DUS1L, RBM5, etc. At the same time, our study also fetched the significant genes with three other COVID-19 gene expression studies [(Xiong et al. (26), Ziegler et al. (27), and Jain et al. (28)] and compared with their expressed genes. The analysis showed the significant genes expressed in other studies are CXCL1, CXCL6, CXCL8, IL33, TIMP1, IL18, IFNGR2, TRIM27, TRIM28, IFI6, XAF1, CXCL5, IFIT1, IL6, IL10, and CSF2, etc. The analysis will help to understand the DEGs in mild and severe COVID-19 patients.

Our protein interactome map analysis found that the number of interactions was 2901, the number of proteins that participated in the interactions was 453, and the average node degree was 12.53. Also, we developed a protein interactome from Xiong et al.’s study using bronchoalveolar lavage fluid. We found that the number of interactions was 76, the number of proteins that participated in the interactions was 35, and the average node degree was 4.06. Similarly, the study from the same authors but with different samples (from the PBMC samples) was used to develop a protein interactome map. The analysis revealed that the number of interactions was 103, and the number of proteins that participated in the interactions was 62. The average number of proteins that participated in the interactions node degree was 3.11.

Similarly, the developed protein interactome from the study of Ziegler et al. informed us the interaction proteins that the number of interactions was 2613, the number of proteins that participated in the interaction was 504, and the average node degree was 10.15. Finally, we developed a protein interactome from the study of Jain et al. We found that the number of interactions was 437, the number of proteins that participated in the interaction was 128, and the average node degree was 6.56. The analysis provides global insights into genome function and cellular organization in COVID-19 patients, indicating interactome networks in COVID-19 patients.

The enrichment cluster analysis of the PPI network showed that the MPHOSPH6, RPL19, EIF3G, EIF3E, RPL1BA, EXOCS5, EXOSC2, RPL3L, RPS3, RPS16, RPL35, EIF4G1, SKIV2L2 firmly formed the central cluster of this PPI network. However, some side clusters were noted associated with the proteins like SKIV2L2, EIF3G, EXOSC2, and RPS16. At the same time, our analysis identified 24 significant lncRNA genes, which will help understand the differentially expressed lncRNA genes and help understand future researchers more about the SARS-CoV-2-infected human cells. Moreover, we found six lncRNAs (TALAM1, DLEU2, and UICLM CASC18, SNHG20, and GNAS) involved in the protein-coding DEG-lncRNA network. This finding is significant for the next-generation biomarker detection point of view.

Finally, the analysis of gene function annotation and categorization of regulated genes related to immune response elements found that several genes are directly or indirectly associated with the inter immunity defense mechanism such as EDN1 MPHOSPH6, RPL19. EDN1 gene is associated with TLR4 response (54). At the same time, RPL19 might be related to the TLR3 receptor-associated signaling and endorses cytokine secretion (101). However, our analysis (our gene and the genes from the other study) found that genes are associated with cytokine up-regulation.

### Findings and Their Direct Implications

The study generated an interactome map, a PPI pattern, a cluster analysis of the PPI network, a pathway enrichment analysis from our research, and other experimental investigations to understand the gene expression and transcriptome profiling of SARS-CoV-2-infected human cells in mild and severe COVID-19 patients. We identified the differentially expressed lncRNA genes of COVID-19 patients and constructed the DEG-lncRNA co-expression networks. We also found six lncRNAs that are involved in the protein-coding DEG-lncRNA network generation. The attempt will help to understand the lncRNA expression in mild and severe COVID-19 patients. These lncRNAs might serve as next-generation potential biomarkers for COVID-19 patients. Presently, one significant objective worldwide is to understand the dysregulation of immune response and inflammation, immunity, and intervention in COVID‐19 patients. We attempted to understand the gene regulation of immune cells and their components and the protective immunity genes using our DEGs data and other COVID-19 studies done in different parts of the world.

### Context of this Study and Other Studies in the Field

Previously, to understand the host transcriptional responses of SARS-CoV-2, an interactome study was performed and was reported by Messina et al. (102). Their study suggested that the host interactome is linked to the S-glycoprotein of the virus mainly *via* the innate immunity machinery, such as cytokines, chemokines, and TLRs. We, too, found in our interactome study that several proteins from COVID-19 patients are linked to innate immunity and the regulation of protective immunity. However, our report is more detailed and unique because our analysis of protein-coding DEGs considers data not only from our group but from many interactome studies from around the globe, and the patient samples from which the data were obtained were diverse. Recently, Gordon et al. prepared an interactome map to understand the protein-protein interactions between human proteins and this virus (103). Simultaneously, Bojkova et al. performed proteomics analysis of host cells infected with SARS-CoV-2. This study revealed new drug targets that could be helpful for drug repurposing to combat this disease (104). The investigation also unfolds new therapeutic targets and will be beneficial for discovering therapeutic.

The construction of the PPI network from patient samples has an immense advantage, which will help to understand disease mechanisms (105–107). Zhang et al. recently developed the SARS-CoV-2 virus-human PPI network using the random walk model to understand pathological biomarkers (108). We have developed a human PPI network from independent studies utilizing samples collected from COVID-19 patients presenting mild and severe symptoms. It comprehensively comprises upregulated protein-coding genes and PPIs in COVID-19 patients from an entire proteome landscape. Enrichment cluster analysis shows densely interlinked regions of proteins as intra-cluster and inter-cluster, which helps us understand densely interlinked regions of proteins in the global proteome landscape and the proteome related to innate immunity and protective immunity in response to SARS-CoV-2 infection. We used the MCODE plug-in from Cytoscape to construct the PPI network and enrichment cluster analysis.

We performed functional pathway enrichment analysis to understand both the COVID-19 regulated genes and the target genes. We report several genes that are regulated in COVID-19 and target genes related to immune cell activation, such as the T- and B-cell-activating protein-coding genes (109). Other researchers have also performed pathway enrichment analysis to understand the lncRNA prognostic signature of ovarian cancer (110). Our functional pathway enrichment analysis may inform us about the significant immune marker genes in COVID-19 patients. Our results also corroborate with the study of Wu et al., who performed functional enrichment analysis to understand the possible role of naïve B cells from the lungs of patients with severe immune responses in COVID-19 patients (111). Our protein-coding DEG-lncRNA co-expression network pattern revealed the prospective function of differentially expressed lncRNAs in the context of COVID-19. Recently, Hu et al. developed co-expression network construction using DEG-lncRNA pairs to understand lncRNAs and proteins in hypertrophic cardiomyopathy (112). Our analysis of protein-coding DEG-lncRNA pairs revealed that six lncRNA have participated in the protein-coding DEG-lncRNA network (TALAM1, DLEU2, UICLM, CASC18, SNHG20, and GNAS). These essential lncRNAs may serve as potential biomarkers for COVID-19. However, further functional studies in a larger cohort of patients need to be investigated.

It is critical to understand the dysregulation of immune response and inflammation, immunity, and intervention in COVID‐19 patients (113) . Recently, Zhou et al. mapped DEGs involved in innate immunity from COVID-19 patients (25). We have annotated and categorized the function of genes related to the regulation of immune elements and protective immunity by the analysis of DEGs in COVID‐19; a list of the immune system’s regulatory genes and regulation of immune-related transcripts in COVID-19 is presented.

### Potential Interpretations of the Study

The potential interpretations of the study can be understood in the following points. Firstly, we know the gene expression and transcriptome profiling of mild and severe COVID-19 patients. Secondly, our analysis of the noted six essential lncRNAs might serve as nest generation biomarkers for COVID-19. However, further functional studies in a larger cohort of patients need to be investigated. Finally, the study prepared a detailed list of the immune system’s regulatory genes and immune-related transcripts in COVID-19, which has immense implications for understanding the COVID-19 dysregulation of immune response and interference in COVID‐19 patients.

### Limitations of the Study

The study suffers from the limitation that the sample size is relatively small due to the available datasets. The dataset we have used for the study (GSE164805) contains fifteen human subjects (five control, five mild COVID-19 patients, and five severe COVID-19 patients). The dataset was submitted by other researchers (100). The data set is limited, as it has been collected only from peripheral blood mononuclear cells (PBMC) samples from three groups of human subjects (control, mild COVID-19 patients, and severe COVID-19 patients). A similar dataset is not available in the database that uses the PBMC for their analysis from COVID-19 patients and compares with control. It was noted that this dataset was the only first dataset in the GEO database which captured the gene expression data from three groups of human subjects and informed differential gene expression of both three groups of patients. The gene expression data was analyzed using a microarray platform. The dataset was initially submitted in the database early (January 2021) when no gene expression data were available from both three groups of COVID-19 patients. In this point of view, it is a very significant dataset. However, as the sample size was small, we compared our result with three other COVID-19 gene expression studies, which were performed by Xiong et al. (26), Ziegler et al. (27), and Jain et al. (28).

## Conclusion

Here, we report the DEG data from COVID-19 patients that will help to understand global gene expression in COVID-19 patients. The data provide valuable information about the immune response in patients infected with SARS-CoV-2, highlighting the molecular genetic mechanisms related to immune elements and protective immunity against COVID-19. We understand that a limited number of patient datasets were analyzed to map the DEGs. However, in the future, we will plan to perform a scRNA-seq study in this direction, which will help better to understand the underlying gene expression mechanism of severe COVID-19 patients compared to mild patients. We believe that similar studies with more patient datasets from other parts of the world will significantly augment our understanding of this complex host-virus interaction during COVID-19 disease progression and will help to map the genes involved in protective immunity.

## Data Availability Statement

The original contributions presented in the study are included in the article/**Supplementary Material**. Further inquiries can be directed to the corresponding authors.

## Author Contributions

CC analyzed and interpreted the patient dataset from the GEO, performed the main experiments, and wrote the main manuscript. ARS performed the data validation, formal analysis, review, and edited of the manuscript. MB performed the data validation and formal analysis. HZ and S-SL performed review and editing of the manuscript. All authors contributed to the article and approved the submitted version.

## Funding

This study was supported by the Hallym University Research Fund and the Basic Science Research Program through the National Research Foundation of Korea (NRF) funded by the Ministry of Education (NRF-2020R1C1C1008694 & NRF-2020R1I1A3074575).

## Conflict of Interest

The authors declare that the research was conducted in the absence of any commercial or financial relationships that could be construed as a potential conflict of interest.

## Publisher’s Note

All claims expressed in this article are solely those of the authors and do not necessarily represent those of their affiliated organizations, or those of the publisher, the editors and the reviewers. Any product that may be evaluated in this article, or claim that may be made by its manufacturer, is not guaranteed or endorsed by the publisher.

## Abbreviations

DEGs, Differentially expressed genes; lncRNA, Long non-coding RNA; SARS-CoV-2, Severe acute respiratory syndrome coronavirus 2; COVID-19, Coronavirus disease; CRP, C-reactive protein; PBMCs, Peripheral blood mononuclear cells; PPI, Protein–protein interaction; UMPA, Uniform manifold approximation and projection; GO, Gene ontology; NCBI, National Center for Biotechnology Information; MD, Mean difference.
