# Supplementary material for: Understanding Gene Expression and Transcriptome Profiling of COVID-19: An Initiative Towards the Mapping of Protective Immunity Genes Against SARS-CoV-2 Infection
Source: Front Immunol. 2021 Dec 15;12:724936. doi: 10.3389/fimmu.2021.724936 (PMC8714830; doi:10.3389/fimmu.2021.724936)
Supplement: Supplementary file 1 [file Table_1.docx]

**Table S1.** Top 250 differentially expressed genes (DEGs) and their ID, P Value, F, gene description

| **ID** | **P.Value** | **F** | **TRANSCRIPT_TYPE** | **GENE.DESCRIPTION** |
| --- | --- | --- | --- | --- |
| ASHGV40004514V5 | 2.60e-15 | 612.8 | lncRNA |  |
| ASHG19AP1B100079683V5 | 4.51e-15 | 569.6 | protein_coding | hyaluronan and proteoglycan link protein 2 [Source:HGNC Symbol;Acc:HGNC:17410] |
| ASHG19LNC1A104444605V5 | 6.88e-15 | 538.5 | lncRNA |  |
| ASHG19LNC1A100061261V5 | 8.75e-15 | 521.6 | lncRNA | query_DPI = chr3:148847330..148847336,-;max_hit_DPIClstrID = chr3:148847397..148847495,+, on up stream;divergent transcription with these coding genes: ENSG00000163755.4 |
| ASHG19AP1B143795176V5 | 1.27e-14 | 496.5 | protein_coding | kallikrein related peptidase 8 [Source:HGNC Symbol;Acc:HGNC:6369] |
| ASHG19LNC1A100069148V5 | 3.89e-14 | 427.7 | lncRNA | query_DPI = chr2:38830303..38830343,+;max_hit_DPIClstrID = chr2:38830030..38830089,-, on up stream;divergent transcription with these coding genes: ENSG00000143889.11 |
| ASHG19LNC1A107586616V5 | 8.14e-14 | 387.6 | lncRNA | novel transcript |
| ASHGV40012850V5 | 1.07e-13 | 373.7 | lncRNA |  |
| ASHG19AP1B130926225V5 | 2.32e-13 | 336.9 | protein_coding |  |
| ASHG19LNC1A100052836V5 | 2.68e-13 | 330.5 | lncRNA |  |
| ASHG19AP1B130179979V5 | 2.83e-13 | 328.1 | protein_coding | dual specificity phosphatase 9 [Source:HGNC Symbol;Acc:HGNC:3076] |
| ASHGV40042878V5 | 2.96e-13 | 326.1 | lncRNA | novel transcript |
| ASHG19AP1B102939844V5 | 4.72e-13 | 306.4 | protein_coding | peroxiredoxin 1 [Source:HGNC Symbol;Acc:HGNC:9352] |
| ASHG19AP1B131783095V5 | 6.58e-13 | 293 | protein_coding | autophagy related 5 [Source:HGNC Symbol;Acc:HGNC:589] |
| ASHG19AP1B106458171V5 | 8.56e-13 | 282.8 | protein_coding | testis expressed 101 [Source:HGNC Symbol;Acc:HGNC:30722] |
| ASHG19LNC1A105945162V5 | 8.93e-13 | 281.2 | lncRNA | KIAA0895 like [Source:HGNC Symbol;Acc:HGNC:34408] |
| ASHG19LNC1A105982028V5 | 1.03e-12 | 275.8 | lncRNA | novel transcript, antisense to MON1A |
| ASHGV40002118V5 | 1.06e-12 | 274.8 | lncRNA | novel transcript |
| ASHG19LNC1A109596949V5 | 1.29e-12 | 267.5 | lncRNA | novel transcript |
| ASHG19LNC1A100048608V5 | 1.33e-12 | 266.6 | lncRNA | novel transcript, antisense DDX52 |
| ASHG19LNC1A101683633V5 | 1.57e-12 | 260.6 | lncRNA | novel transcript |
| ASHG19AP1B101133016V5 | 1.90e-12 | 254.1 | protein_coding | brain expressed associated with NEDD4 1 [Source:HGNC Symbol;Acc:HGNC:24160] |
| ASHG19AP1B127682210V5 | 2.21e-12 | 248.8 | protein_coding | Rho guanine nucleotide exchange factor 1 [Source:HGNC Symbol;Acc:HGNC:681] |
| ASHG19AP1B122326814V5 | 2.50e-12 | 244.8 | protein_coding | tripartite motif containing 6 [Source:HGNC Symbol;Acc:HGNC:16277] |
| ASHG19AP1B101848137V5 | 2.80e-12 | 241.1 | protein_coding | coagulation factor X [Source:HGNC Symbol;Acc:HGNC:3528] |
| ASHG19AP1B100026912V5 | 3.64e-12 | 232.7 | protein_coding | transmembrane protein 252 [Source:HGNC Symbol;Acc:HGNC:28537] |
| ASHG19AP1B100890801V5 | 4.83e-12 | 223.9 | protein_coding | septin 12 [Source:HGNC Symbol;Acc:HGNC:26348] |
| ASHG19AP1B136937830V5 | 5.06e-12 | 222.5 | protein_coding | intraflagellar transport 52 [Source:HGNC Symbol;Acc:HGNC:15901] |
| ASHG19LNC1A109148624V5 | 5.43e-12 | 220.4 | lncRNA | LIPE antisense RNA 1 [Source:HGNC Symbol;Acc:HGNC:48589] |
| ASHG19LNC1A100854911V5 | 6.08e-12 | 217 | lncRNA | long intergenic non-protein coding RNA 2092 [Source:HGNCSymbol;Acc:HGNC:52943] |
| ASHG19LNC1A102103098V5 | 6.56e-12 | 214.8 | lncRNA | query_DPI = chr16:52581132..52581180,+;max_hit_DPIClstrID = chr16:52580953..52580992,-, on up stream;divergent transcription with these coding genes: ENSG00000103460.12 |
| ASHGV40040540V5 | 7.29e-12 | 211.8 | lncRNA |  |
| ASHG19LNC1A100084701V5 | 8.06e-12 | 208.9 | lncRNA | novel transcript |
| ASHG19LNC1A100048539V5 | 8.20e-12 | 208.4 | lncRNA | novel transcript, antisense PRKCZ |
| ASHG19AP1B141920576V5 | 1.36e-11 | 194.5 | protein_coding | S100 calcium binding protein A16 [Source:HGNC Symbol;Acc:HGNC:20441] |
| ASHG19LNC1A110630287V5 | 1.44e-11 | 193.1 | lncRNA | novel transcript, antisense to MON1A |
| ASHG19LNC1A100015590V5 | 1.52e-11 | 191.7 | lncRNA | novel transcript |
| ASHG19LNC1A106045150V5 | 1.69e-11 | 188.9 | lncRNA | FOXF1 adjacent non-coding developmental regulatory RNA [Source:HGNC Symbol;Acc:HGNC:43894] |
| ASHGV40006178V5 | 2.10e-11 | 183.4 | lncRNA |  |
| ASHG19AP1B113236502V5 | 2.17e-11 | 182.6 | protein_coding | zinc finger protein 706 [Source:HGNC Symbol;Acc:HGNC:24992] |
| ASHG19LNC1A102429018V5 | 2.24e-11 | 181.7 | lncRNA | uncharacterized LOC101929753 [Source:NCBI gene;Acc:101929753] |
| ASHG19LNC1A100092968V5 | 2.72e-11 | 177 | lncRNA | novel transcript |
| ASHGV40001413V5 | 2.74e-11 | 176.8 | lncRNA | NA |
| ASHG19LNC1A106399525V5 | 2.78e-11 | 176.5 | lncRNA | novel transcript |
| ASHG19LNC1A100063734V5 | 2.91e-11 | 175.4 | lncRNA | uncharacterized LOC105376072 [Source:NCBI gene;Acc:105376072] |
| ASHGV40010017V5 | 3.12e-11 | 173.7 | lncRNA | HOXC13 antisense RNA [Source:HGNC Symbol;Acc:HGNC:43753] |
| ASHG19LNC1A100076220V5 | 3.28e-11 | 172.5 | lncRNA | novel transcript |
| ASHG19AP1B126722368V5 | 3.35e-11 | 172 | protein_coding | peroxisomal membrane protein 4 [Source:HGNC Symbol;Acc:HGNC:15920] |
| ASHG19LNC1A100052346V5 | 3.60e-11 | 170.4 | lncRNA | NKX2-2 antisense RNA 1 [Source:HGNC Symbol;Acc:HGNC:37154] |
| ASHG19LNC1A104465952V5 | 3.72e-11 | 169.6 | lncRNA | ADIRF antisense RNA 1 [Source:HGNC Symbol;Acc:HGNC:45127] |
| ASHGV40059791V5 | 3.74e-11 | 169.5 | lncRNA |  |
| ASHG19AP1B116753425V5 | 4.03e-11 | 167.8 | protein_coding | ZFP37 zinc finger protein [Source:HGNC Symbol;Acc:HGNC:12863] |
| ASHGV40053220V5 | 4.23e-11 | 166.6 | lncRNA |  |
| ASHG19AP1B139781923V5 | 4.35e-11 | 166 | protein_coding | immunoglobulin like domain containing receptor 1 [Source:HGNC Symbol;Acc:HGNC:28741] |
| ASHG19LNC1A100020163V5 | 4.39e-11 | 165.8 | lncRNA | novel transcript |
| ASHG19LNC1A104893279V5 | 4.59e-11 | 164.8 | lncRNA | semaphorin 3B [Source:HGNC Symbol;Acc:HGNC:10724] |
| ASHG19AP1B124346093V5 | 4.70e-11 | 164.3 | protein_coding | SP140 nuclear body protein [Source:HGNC Symbol;Acc:HGNC:17133] |
| ASHG19AP1B100227479V5 | 4.85e-11 | 163.6 | protein_coding | hes family bHLH transcription factor 5 [Source:HGNC Symbol;Acc:HGNC:19764] |
| ASHGV40057731V5 | 5.25e-11 | 161.8 | lncRNA | MIR210 host gene [Source:HGNC Symbol;Acc:HGNC:39524] |
| ASHG19LNC1A107625614V5 | 5.67e-11 | 160.1 | lncRNA | novel transcript |
| ASHG19LNC1A101637126V5 | 5.71e-11 | 159.9 | lncRNA | deleted in lymphocytic leukemia 2 [Source:HGNC Symbol;Acc:HGNC:13748] |
| ASHG19LNC1A110416853V5 | 6.26e-11 | 157.9 | lncRNA | novel transcript |
| ASHG19LNC1A100080479V5 | 6.53e-11 | 157 | lncRNA | MEF2C antisense RNA 2 [Source:HGNC Symbol;Acc:HGNC:53115] |
| ASHG19AP1B141555700V5 | 7.58e-11 | 153.8 | protein_coding | MARCKS like 1 [Source:HGNC Symbol;Acc:HGNC:7142] |
| ASHGV40026229V5 | 7.81e-11 | 153.2 | lncRNA |  |
| ASHG19LNC1A100076699V5 | 7.92e-11 | 152.9 | lncRNA | novel transcript, antisense to PRSS8 |
| ASHG19AP1B121660446V5 | 8.05e-11 | 152.5 | protein_coding | uromodulin [Source:HGNC Symbol;Acc:HGNC:12559] |
| ASHG19LNC1A100032732V5 | 8.33e-11 | 151.8 | lncRNA | novel transcript, antisense to ADGRV1 |
| ERCC-00097_63 | 8.76e-11 | 150.8 |  |  |
| ASHGV40050778V5 | 9.00e-11 | 150.2 | lncRNA |  |
| ASHG19LNC1A101637125V5 | 9.00e-11 | 150.2 | lncRNA | deleted in lymphocytic leukemia 2 [Source:HGNC Symbol;Acc:HGNC:13748] |
| ASHGV40059259V5 | 9.14e-11 | 149.9 | lncRNA |  |
| ASHG19AP1B100102680V5 | 9.99e-11 | 148.1 | protein_coding | transmembrane protein 151A [Source:HGNC Symbol;Acc:HGNC:28497] |
| ASHG19AP1B140228294V5 | 1.02e-10 | 147.7 | protein_coding | NDUFA4, mitochondrial complex associated like 2 [Source:HGNC Symbol;Acc:HGNC:29836] |
| ASHG19LNC1A102825717V5 | 1.08e-10 | 146.4 | lncRNA | uncharacterized LOC101929753 [Source:NCBI gene;Acc:101929753] |
| ASHG19AP1B127764973V5 | 1.13e-10 | 145.7 | protein_coding | proteasome inhibitor subunit 1 [Source:HGNC Symbol;Acc:HGNC:9571] |
| ASHG19LNC1A109221690V5 | 1.16e-10 | 145.1 | lncRNA | novel transcript, antisense to C18orf34 |
| ASHG19LNC1A108946762V5 | 1.20e-10 | 144.4 | lncRNA |  |
| ASHG19LNC1A103685577V5 | 1.24e-10 | 143.8 | lncRNA | query_DPI = chr11:122933335..122933379,+;max_hit_DPIClstrID = chr11:122932835..122932852,-, on up stream;divergent transcription with these coding genes: ENSG00000109971.9 |
| ASHG19LNC1A100032333V5 | 1.31e-10 | 142.7 | lncRNA | novel transcript, overlapping NRARP |
| ASHG19LNC1A100076703V5 | 1.35e-10 | 142 | lncRNA | novel transcript, overlapping to GCM1 |
| ASHG19AP1B105392872V5 | 1.37e-10 | 141.8 | protein_coding | ribosomal protein L3 like [Source:HGNC Symbol;Acc:HGNC:10351] |
| ASHG19AP1B108131779V5 | 1.44e-10 | 140.8 | protein_coding | stabilizer of axonemal microtubules 2 [Source:HGNC Symbol;Acc:HGNC:33727] |
| ASHG19AP1B109420977V5 | 1.53e-10 | 139.6 | protein_coding | At least 1 of the 10 top ranked transcript is CPAT coding and max ORF size >=300, NOT annotated as nonCoding in GENCODE |
| ASHG19AP1B102211524V5 | 1.55e-10 | 139.4 | protein_coding | carboxypeptidase A3 [Source:HGNC Symbol;Acc:HGNC:2298] |
| ASHGV40059449V5 | 1.59e-10 | 138.9 | lncRNA |  |
| ASHGV40001229V5 | 1.70e-10 | 137.6 | lncRNA | uncharacterized LOC200772 [Source:NCBI gene;Acc:200772] |
| ASHG19AP1B100262006V5 | 1.87e-10 | 135.9 | protein_coding | gap junction protein delta 3 [Source:HGNC Symbol;Acc:HGNC:19147] |
| ASHG19AP1B100226035V5 | 1.92e-10 | 135.3 | protein_coding | interferon regulatory factor 2 binding protein 1 [Source:HGNC Symbol;Acc:HGNC:21728] |
| ASHG19AP1B116099560V5 | 1.95e-10 | 135 | protein_coding | ceramide kinase like [Source:HGNC Symbol;Acc:HGNC:21699] |
| ASHG19AP1B119162740V5 | 2.14e-10 | 133.3 | protein_coding | ribosomal protein S3 [Source:HGNC Symbol;Acc:HGNC:10420] |
| ASHG19LNC1A109158522V5 | 2.15e-10 | 133.2 | lncRNA | long intergenic non-protein coding RNA 1389 [Source:HGNC Symbol;Acc:HGNC:50661] |
| ASHG19LNC1A100454332V5 | 2.16e-10 | 133.1 | lncRNA | long intergenic non-protein coding RNA 1198 [Source:HGNCSymbol;Acc:HGNC:49598] |
| ASHGV40001197V5 | 2.30e-10 | 131.9 | lncRNA | novel transcript |
| ASHG19LNC1A100073073V5 | 2.41e-10 | 131.1 | lncRNA | query_DPI = chr12:12420263..12420313,+;max_hit_DPIClstrID = chr12:12419905..12419989,-, on up stream;divergent transcription with these coding genes: ENSG00000070018.4 |
| ASHG19LNC1A102092106V5 | 2.49e-10 | 130.6 | lncRNA | ADIRF antisense RNA 1 [Source:HGNC Symbol;Acc:HGNC:45127] |
| ASHG19LNC1A100011085V5 | 2.49e-10 | 130.5 | lncRNA | SLCO4A1 antisense RNA 1 [Source:HGNC Symbol;Acc:HGNC:40537] |
| ASHG19LNC1A111689598V5 | 2.50e-10 | 130.4 | lncRNA | TBC1 domain family member 17 [Source:HGNC Symbol;Acc:HGNC:25699] |
| ASHG19LNC1A107535210V5 | 2.60e-10 | 129.8 | lncRNA | FAM167A antisense RNA 1 [Source:HGNC Symbol;Acc:HGNC:15548] |
| ASSPINKEIN100003643 | 2.80e-10 | 128.5 |  |  |
| ASHG19AP1B104673574V5 | 2.96e-10 | 127.5 | protein_coding | chromosome 3 open reading frame 80 [Source:HGNC Symbol;Acc:HGNC:40048] |
| ASHG19LNC1A100093030V5 | 3.29e-10 | 125.6 | lncRNA | novel transcript, antisense to AMN1 |
| ASHG19AP1B115327726V5 | 3.32e-10 | 125.4 | protein_coding | tubby like protein 2 [Source:HGNC Symbol;Acc:HGNC:12424] |
| ASHGV40051007V5 | 3.38e-10 | 125.1 | lncRNA | uncharacterized LOC105375671 [Source:NCBI gene;Acc:105375671] |
| ASHGV40052827V5 | 3.42e-10 | 124.9 | lncRNA |  |
| ASHGV40033809V5 | 3.58e-10 | 124.1 | lncRNA | long intergenic non-protein coding RNA 1639 [Source:HGNC Symbol;Acc:HGNC:52426] |
| ASHG19LNC1A104002307V5 | 3.68e-10 | 123.6 | lncRNA | differentiation antagonizing non-protein coding RNA [Source:HGNC Symbol;Acc:HGNC:28964] |
| ASHG19AP1B118570399V5 | 3.73e-10 | 123.4 | protein_coding | RNA binding motif protein 5 [Source:HGNC Symbol;Acc:HGNC:9902] |
| ASHG19AP1B102926930V5 | 3.73e-10 | 123.4 | protein_coding | fibroblast growth factor 1 [Source:HGNC Symbol;Acc:HGNC:3665] |
| ASHGV40040841V5 | 3.86e-10 | 122.8 | lncRNA | long intergenic non-protein coding RNA 1959 [Source:HGNC Symbol;Acc:HGNC:27965] |
| ASHG19LNC1A100048727V5 | 3.88e-10 | 122.8 | lncRNA |  |
| ASHG19AP1B100010099V5 | 3.95e-10 | 122.4 | protein_coding | At least 1 of the 10 top ranked transcript is CPAT coding and max ORF size >=300, NOT annotated as nonCoding in GENCODE |
| ASHGV40016060V5 | 3.95e-10 | 122.4 | lncRNA |  |
| ASHG19AP1B100143862V5 | 4.12e-10 | 121.7 | protein_coding | endothelin 1 [Source:HGNC Symbol;Acc:HGNC:3176] |
| ASHG19AP1B127343616V5 | 4.24e-10 | 121.2 | protein_coding | spalt like transcription factor 2 [Source:HGNC Symbol;Acc:HGNC:10526] |
| ASHG19AP1B141086300V5 | 4.38e-10 | 120.7 | protein_coding | mutY DNA glycosylase [Source:HGNC Symbol;Acc:HGNC:7527] |
| ASHG19AP1B133251296V5 | 4.56e-10 | 120 | protein_coding | MAGE family member A10 [Source:HGNC Symbol;Acc:HGNC:6797] |
| ASHG19LNC1A107787072V5 | 4.65e-10 | 119.7 | lncRNA | MDM2 proto-oncogene [Source:HGNC Symbol;Acc:HGNC:6973] |
| ASHG19LNC1A100052843V5 | 4.66e-10 | 119.6 | lncRNA |  |
| ASHG19AP1B116251415V5 | 4.84e-10 | 119 | protein_coding | At least 1 of the 10 top ranked transcript is CPAT coding and max ORF size >=300, NOT annotated as nonCoding in GENCODE |
| ASHGV40028330V5 | 4.96e-10 | 118.6 | lncRNA | uncharacterized LOC200772 [Source:NCBI gene;Acc:200772] |
| ASHG19LNC1A108788791V5 | 5.13e-10 | 118.1 | lncRNA | NDUFA6 divergent transcript [Source:HGNCSymbol;Acc:HGNC:45273] |
| ASHG19AP1B100207377V5 | 5.15e-10 | 118 | protein_coding | tigger transposable element derived 3 [Source:HGNC Symbol;Acc:HGNC:18334] |
| ASHG19LNC1A109555842V5 | 5.23e-10 | 117.7 | lncRNA | testis associated oncogenic lncRNA [Source:HGNC Symbol;Acc:HGNC:53788] |
| ASHG19LNC1A100034530V5 | 5.23e-10 | 117.7 | lncRNA | ankyrin repeat domain 30B like [Source:HGNC Symbol;Acc:HGNC:35167] |
| ASHGV40030424V5 | 5.49e-10 | 116.9 | lncRNA | NRSN2 antisense RNA 1 [Source:HGNC Symbol;Acc:HGNC:51222] |
| ASHGV40034868V5 | 5.88e-10 | 115.8 | lncRNA |  |
| ASHG19AP1B105033128V5 | 5.94e-10 | 115.7 | protein_coding | striatin 4 [Source:HGNC Symbol;Acc:HGNC:15721] |
| ASHG19LNC1A109552992V5 | 6.07e-10 | 115.3 | lncRNA |  |
| ASHG19LNC1A100072148V5 | 6.33e-10 | 114.6 | lncRNA | novel transcript |
| ASHG19AP1B102268874V5 | 6.61e-10 | 114 | protein_coding | kinesin family member 27 [Source:HGNC Symbol;Acc:HGNC:18632] |
| ASHG19LNC1A100004110V5 | 6.61e-10 | 114 | lncRNA |  |
| ASHG19LNC1A100057076V5 | 6.68e-10 | 113.8 | lncRNA | long non-coding RNA and at least 2000 nt from coding related gene on both strand;standalone;mono_exonic |
| ASHG19LNC1A110290204V5 | 6.75e-10 | 113.6 | lncRNA | small nucleolar RNA host gene 20 [Source:HGNC Symbol;Acc:HGNC:33099] |
| ASHGV40029603V5 | 6.81e-10 | 113.5 | lncRNA | ZEB2 antisense RNA 1 [Source:HGNC Symbol;Acc:HGNC:37149] |
| ASHG19LNC1ABL100000566V5 | 6.81e-10 | 113.5 | lncRNA |  |
| ASHG19AP1B108733567V5 | 6.82e-10 | 113.5 | protein_coding | Cdc42 guanine nucleotide exchange factor 9 [Source:HGNCSymbol;Acc:HGNC:14561] |
| ASHG19LNC1A100887318V5 | 6.93e-10 | 113.2 | lncRNA | small nucleolar RNA host gene 1 [Source:HGNC Symbol;Acc:HGNC:32688] |
| ASHG19AP1B106402435V5 | 7.01e-10 | 113 | protein_coding | leucine rich repeat containing 61 [Source:HGNC Symbol;Acc:HGNC:21704] |
| ASHG19LNC1A100089080V5 | 7.15e-10 | 112.7 | lncRNA | novel transcript |
| ASHG19LNC1A110321113V5 | 7.29e-10 | 112.4 | lncRNA | GNAS complex locus [Source:HGNC Symbol;Acc:HGNC:4392] |
| ASSPINKEIN100008173 | 7.33e-10 | 112.3 |  |  |
| ASHG19AP1B100077547V5 | 7.36e-10 | 112.3 | protein_coding | troponin C2, fast skeletal type [Source:HGNC Symbol;Acc:HGNC:11944] |
| ASHG19AP1B135988649V5 | 7.47e-10 | 112 | protein_coding | ER membrane protein complex subunit 6 [Source:HGNC Symbol;Acc:HGNC:28430] |
| ASHG19LNC1A100093161V5 | 7.57e-10 | 111.8 | lncRNA | query_DPI = chr10:70883631..70883685,-;max_hit_DPIClstrID = chr10:70883923..70883997,+, on up stream;divergent transcription with these coding genes: ENSG00000122958.10 |
| ASHG19LNC1A100061364V5 | 7.70e-10 | 111.6 | lncRNA | long non-coding RNA and at least 2000 nt from coding related gene on both strand;standalone;mono_exonic |
| ASHG19LNC1A110168759V5 | 7.73e-10 | 111.5 | lncRNA |  |
| ASHG19AP1B100095122V5 | 8.31e-10 | 110.4 | protein_coding | At least 1 of the 10 top ranked transcript is CPAT coding and max ORF size >=300, NOT annotated as nonCoding in GENCODE |
| ASHG19LNC1A108801755V5 | 8.32e-10 | 110.4 | lncRNA | SRD5A3 antisense RNA 1 [Source:HGNC Symbol;Acc:HGNC:44138] |
| ASHG19LNC1A100044793V5 | 8.36e-10 | 110.3 | lncRNA | novel transcript |
| ASHG19LNC1A112100487V5 | 8.58e-10 | 109.9 | lncRNA | MKLN1 antisense RNA [Source:HGNC Symbol;Acc:HGNC:40374] |
| ASHG19LNC1A100835893V5 | 8.64e-10 | 109.8 | lncRNA | long intergenic non-protein coding RNA 408 [Source:HGNC Symbol;Acc:HGNC:42740] |
| ASHG19AP1B127249610V5 | 8.73e-10 | 109.6 | protein_coding | family with sequence similarity 189 member B [Source:HGNC Symbol;Acc:HGNC:1233] |
| ASHG19LNC1A107590865V5 | 8.75e-10 | 109.6 | lncRNA | novel transcript |
| ASHG19AP1B102078045V5 | 9.07e-10 | 109 | protein_coding | cyclin and CBS domain divalent metal cation transport mediator 2 [Source:HGNC Symbol;Acc:HGNC:103] |
| ASHG19LNC1A100076086V5 | 9.23e-10 | 108.8 | lncRNA | novel transcript |
| ASHG19AP1B100171364V5 | 9.50e-10 | 108.3 | protein_coding | At least 1 of the 10 top ranked transcript is CPAT coding and max ORF size >=300, NOT annotated as nonCoding in GENCODE |
| ASHG19LNC1A106002067V5 | 9.67e-10 | 108.1 | lncRNA | long intergenic non-protein coding RNA 1119 [Source:HGNC Symbol;Acc:HGNC:49262] |
| ASHG19AP1B118160980V5 | 9.85e-10 | 107.8 | protein_coding | UBX domain protein 1 [Source:HGNC Symbol;Acc:HGNC:18402] |
| ASHG19LNC1A100012534V5 | 1.00e-09 | 107.5 | lncRNA | query_DPI = chr1:116519452..116519478,-;max_hit_DPIClstrID = chr1:116519112..116519204,+, on dnstream;divergent transcription with these coding genes: ENSG00000163393.8 |
| ASHG19AP1B100044033V5 | 1.01e-09 | 107.5 | protein_coding | transmembrane protein 143 [Source:HGNC Symbol;Acc:HGNC:25603] |
| ASHG19LNC1A101254421V5 | 1.01e-09 | 107.4 | lncRNA | long intergenic non-protein coding RNA 1358 [Source:HGNCSymbol;Acc:HGNC:50589] |
| ASHG19AP1B101393687V5 | 1.04e-09 | 107 | protein_coding | BTB domain containing 19 [Source:HGNC Symbol;Acc:HGNC:27145] |
| ASHG19LNC1A106434347V5 | 1.04e-09 | 106.9 | lncRNA | uncharacterized LOC105370854 [Source:NCBI gene;Acc:105370854] |
| ASHG19LNC1A112379221V5 | 1.11e-09 | 105.9 | lncRNA | Era like 12S mitochondrial rRNA chaperone 1 [Source:HGNC Symbol;Acc:HGNC:3424] |
| ASHG19AP1B132472269V5 | 1.16e-09 | 105.3 | protein_coding | RAS protein activator like 2 [Source:HGNC Symbol;Acc:HGNC:9874] |
| ASHG19LNC1A100057683V5 | 1.19e-09 | 104.9 | lncRNA | SEC14 like lipid binding 2 [Source:HGNC Symbol;Acc:HGNC:10699] |
| ASHGV40051914V5 | 1.20e-09 | 104.8 | lncRNA |  |
| ASHG19LNC1A100075651V5 | 1.21e-09 | 104.7 | lncRNA | novel transcript |
| ASHG19AP1B104650775V5 | 1.24e-09 | 104.4 | protein_coding | carbohydrate sulfotransferase 1 [Source:HGNC Symbol;Acc:HGNC:1969] |
| ASHG19LNC1A110644885V5 | 1.24e-09 | 104.4 | lncRNA | novel transcript, antisense ARHGDIA |
| ASHG19LNC1A101660177V5 | 1.25e-09 | 104.2 | lncRNA | long intergenic non-protein coding RNA 1206 [Source:HGNC Symbol;Acc:HGNC:49637] |
| ASHG19AP1B124077767V5 | 1.25e-09 | 104.2 | protein_coding | RalGTPase activating protein catalytic alpha subunit 2 [Source:HGNC Symbol;Acc:HGNC:16207] |
| ASHG19AP1B117393755V5 | 1.30e-09 | 103.6 | protein_coding | At least 1 of the 10 top ranked transcript is CPAT coding and max ORF size >=300, NOT annotated as nonCoding in GENCODE |
| ASHGV40004388V5 | 1.32e-09 | 103.5 | lncRNA |  |
| ASHG19AP1B120812793V5 | 1.34e-09 | 103.2 | protein_coding | At least 1 of the 10 top ranked transcript is CPAT coding and max ORF size >=300, NOT annotated as nonCoding in GENCODE |
| ASHG19AP1B110829606V5 | 1.35e-09 | 103.1 | protein_coding | transmembrane protein 106B [Source:HGNC Symbol;Acc:HGNC:22407] |
| ASHG19LNC1A100028094V5 | 1.41e-09 | 102.5 | lncRNA | novel transcript |
| ASHG19LNC1A106532476V5 | 1.42e-09 | 102.3 | lncRNA | sperm associated antigen 1 [Source:HGNC Symbol;Acc:HGNC:11212] |
| ASHG19AP1B100140590V5 | 1.45e-09 | 102.1 | protein_coding | MOS proto-oncogene, serine/threonine kinase [Source:HGNC Symbol;Acc:HGNC:7199] |
| ASHG19AP1B104000725V5 | 1.46e-09 | 102 | protein_coding | pancreatic polypeptide [Source:HGNC Symbol;Acc:HGNC:9327] |
| ASHGV40023539V5 | 1.46e-09 | 102 | lncRNA | long intergenic non-protein coding RNA 2582 [Source:HGNC Symbol;Acc:HGNC:53792] |
| ASHG19LNC1A102866939V5 | 1.47e-09 | 101.9 | lncRNA | novel transcript |
| ASHG19LNC1A106013140V5 | 1.47e-09 | 101.9 | lncRNA |  |
| ASHGV40048341V5 | 1.48e-09 | 101.7 | lncRNA | long intergenic non-protein coding RNA 1393 [Source:HGNC Symbol;Acc:HGNC:50669] |
| ASHG19AP1B133038450V5 | 1.49e-09 | 101.7 | protein_coding | dihydrouridine synthase 1 like [Source:HGNC Symbol;Acc:HGNC:30086] |
| ASHGV40021624V5 | 1.50e-09 | 101.6 | lncRNA |  |
| ASHG19LNC1A102085612V5 | 1.51e-09 | 101.5 | lncRNA | novel transcript |
| ASHG19LNC1A102428297V5 | 1.52e-09 | 101.4 | lncRNA | deleted in lymphocytic leukemia 2 [Source:HGNC Symbol;Acc:HGNC:13748] |
| ASHG19AP1B101019195V5 | 1.54e-09 | 101.2 | protein_coding | LON peptidase N-terminal domain and ring finger 1 [Source:HGNCSymbol;Acc:HGNC:26302] |
| ASHG19LNC1A100088080V5 | 1.56e-09 | 101 | lncRNA | novel transcript |
| ASHG19LNC1A100482211V5 | 1.56e-09 | 101 | lncRNA | lung cancer associated transcript 1 [Source:HGNC Symbol;Acc:HGNC:48498] |
| ASHG19AP1B100197615V5 | 1.58e-09 | 100.8 | protein_coding | toll like receptor 6 [Source:HGNC Symbol;Acc:HGNC:16711] |
| ASHG19LNC1A100072825V5 | 1.64e-09 | 100.3 | lncRNA | novel transcript |
| ASHG19LNC1A106475763V5 | 1.67e-09 | 100.1 | lncRNA | carnitine O-acetyltransferase [Source:HGNC Symbol;Acc:HGNC:2342] |
| ASHG19LNC1A105245011V5 | 1.67e-09 | 100.1 | lncRNA | novel transcript, antisense to KCNC2 |
| ASHG19LNC1A100012092V5 | 1.68e-09 | 100 | lncRNA | novel transcript |
| ASHG19AP1B143197067V5 | 1.68e-09 | 100 | protein_coding | adaptor related protein complex 2 subunit mu 1 [Source:HGNC Symbol;Acc:HGNC:564] |
| ASHG19LNC1A102812538V5 | 1.74e-09 | 99.5 | lncRNA | long intergenic non-protein coding RNA 1876 [Source:HGNC Symbol;Acc:HGNC:52695] |
| ASHG19LNC1ABL100000870V5 | 1.76e-09 | 99.3 | lncRNA |  |
| ASHG19AP1B111702966V5 | 1.78e-09 | 99.2 | protein_coding | At least 1 of the 10 top ranked transcript is CPAT coding and max ORF size >=300, NOT annotated as nonCoding in GENCODE |
| ASHG19LNC1A100084341V5 | 1.82e-09 | 98.8 | lncRNA | INTS6 antisense RNA 1 [Source:HGNC Symbol;Acc:HGNC:42691] |
| ASHGV40019012V5 | 1.84e-09 | 98.7 | lncRNA |  |
| ASHG19AP1B108818018V5 | 1.88e-09 | 98.4 | protein_coding | tropomodulin 3 [Source:HGNC Symbol;Acc:HGNC:11873] |
| ASHG19LNC1A106039798V5 | 1.89e-09 | 98.3 | lncRNA | novel transcript, antisense to PSMD7 |
| ASHG19LNC1ABL100000681V5 | 1.89e-09 | 98.3 | lncRNA |  |
| ASHG19AP1B107974207V5 | 1.91e-09 | 98.2 | protein_coding | LLLL and CFNLAS motif containing 1 [Source:HGNC Symbol;Acc:HGNC:21750] |
| ASHGV40024194V5 | 1.93e-09 | 98 | lncRNA |  |
| ASHG19AP1B102930566V5 | 1.94e-09 | 97.9 | protein_coding | eukaryotic translation initiation factor 4 gamma 1 [Source:HGNC Symbol;Acc:HGNC:3296] |
| ASHG19LNC1A100060784V5 | 1.98e-09 | 97.7 | lncRNA | novel transcript |
| ASHG19AP1B124609586V5 | 2.01e-09 | 97.5 | protein_coding | signal induced proliferation associated 1 like 2 [Source:HGNC Symbol;Acc:HGNC:23800] |
| ASHG19AP1B101292487V5 | 2.03e-09 | 97.3 | protein_coding | smoothelin [Source:HGNC Symbol;Acc:HGNC:11126] |
| ASHGV40037100V5 | 2.05e-09 | 97.2 | lncRNA | uncharacterized LOC101928521 [Source:NCBI gene;Acc:101928521] |
| ASHG19LNC1A109427515V5 | 2.06e-09 | 97.1 | lncRNA | OBSCN antisense RNA 1 [Source:HGNC Symbol;Acc:HGNC:32047] |
| ASHG19AP1B100785967V5 | 2.09e-09 | 97 | protein_coding | ubiquitin specific peptidase 44 [Source:HGNC Symbol;Acc:HGNC:20064] |
| ASHG19AP1B142926223V5 | 2.13e-09 | 96.7 | protein_coding | cAMP responsive element binding protein 3 like 2 [Source:HGNC Symbol;Acc:HGNC:23720] |
| ASHG19LNC1A108832336V5 | 2.15e-09 | 96.5 | lncRNA | novel transcript |
| ASHG19AP1B103461095V5 | 2.17e-09 | 96.4 | protein_coding | keratin 2 [Source:HGNC Symbol;Acc:HGNC:6439] |
| ASHGV40029368V5 | 2.19e-09 | 96.3 | lncRNA | long intergenic non-protein coding RNA 1191 [Source:HGNC Symbol;Acc:HGNC:49595] |
| ASHG19LNC1A104876954V5 | 2.19e-09 | 96.3 | lncRNA | long non-coding RNA and at least 2000 nt from coding related gene on both strand;with antisense partner;multi_exonic |
| ASHG19LNC1A109294555V5 | 2.31e-09 | 95.6 | lncRNA | ankyrin repeat domain 13A [Source:HGNC Symbol;Acc:HGNC:21268] |
| ASHG19LNC1A100028099V5 | 2.32e-09 | 95.5 | lncRNA | novel transcript |
| ASHGV40051026V5 | 2.33e-09 | 95.4 | lncRNA | UBR5 antisense RNA 1 [Source:HGNC Symbol;Acc:HGNC:51661] |
| ASHG19LNC1ABL100000390V5 | 2.40e-09 | 95.1 | lncRNA |  |
| ASHG19LNC1A108131956V5 | 2.46e-09 | 94.7 | lncRNA | armadillo repeat containing 7 [Source:HGNC Symbol;Acc:HGNC:26168] |
| ASHG19AP1B100219166V5 | 2.46e-09 | 94.7 | protein_coding | At least 1 of the 10 top ranked transcript is CPAT coding and max ORF size >=300, NOT annotated as nonCoding in GENCODE |
| ASHG19AP1B134807541V5 | 2.47e-09 | 94.7 | protein_coding | GCSAML antisense RNA 1 [Source:HGNC Symbol;Acc:HGNC:41244] |
| ASHG19AP1B124487458V5 | 2.48e-09 | 94.6 | protein_coding | male germ cell associated kinase [Source:HGNC Symbol;Acc:HGNC:6816] |
| ASHG19AP1B116871577V5 | 2.50e-09 | 94.5 | protein_coding | tubulin folding cofactor D [Source:HGNC Symbol;Acc:HGNC:11581] |
| ASHGV40050040V5 | 2.51e-09 | 94.5 | lncRNA |  |
| ASHG19LNC1A100043936V5 | 2.59e-09 | 94 | lncRNA | novel transcript, antisense to ADAMTSL4 |
| ASHG19LNC1A100024725V5 | 2.61e-09 | 94 | lncRNA | long non-coding RNA and at least 2000 nt from coding related gene on both strand;with antisense partner;mono_exonic |
| ASHG19LNC1A109170827V5 | 2.63e-09 | 93.9 | lncRNA | uncharacterized LOC100131047 [Source:NCBI gene;Acc:100131047] |
| ASHG19AP1B127674344V5 | 2.63e-09 | 93.8 | protein_coding | protein phosphatase 1 regulatory subunit 12A [Source:HGNCSymbol;Acc:HGNC:7618] |
| ASHG19AP1B112128398V5 | 2.65e-09 | 93.7 | protein_coding | potassium voltage-gated channel subfamily J member 2 [Source:HGNC Symbol;Acc:HGNC:6263] |
| ASHG19LNC1ABL100000341V5 | 2.68e-09 | 93.6 | lncRNA |  |
| ASHG19AP1B114603136V5 | 2.70e-09 | 93.5 | protein_coding | lipocalin 9 [Source:HGNC Symbol;Acc:HGNC:17442] |
| ASHG19LNC1A107597508V5 | 2.71e-09 | 93.4 | lncRNA | NEDD4 binding protein 2 like 2 [Source:HGNC Symbol;Acc:HGNC:26916] |
| ASHG19LNC1A106435265V5 | 2.72e-09 | 93.4 | lncRNA | novel transcript |
| ASHG19LNC1A100025009V5 | 2.77e-09 | 93.1 | lncRNA | long non-coding RNA proximal to ENSG00000117394.15_in_AS_strand;with antisense partner;mono_exonic |
| ASHGV40027552V5 | 2.78e-09 | 93.1 | lncRNA | long intergenic non-protein coding RNA 1920 [Source:HGNC Symbol;Acc:HGNC:52738] |
| ASHG19LNC1A100048535V5 | 2.84e-09 | 92.8 | lncRNA | novel transcript |
| ASHG19AP1B100132859V5 | 2.84e-09 | 92.8 | protein_coding | transmembrane protein 114 [Source:HGNC Symbol;Acc:HGNC:33227] |
| ASHG19AP1B140078097V5 | 2.86e-09 | 92.7 | protein_coding | exosome component 2 [Source:HGNC Symbol;Acc:HGNC:17097] |
| ASHG19LNC1A100032095V5 | 2.89e-09 | 92.6 | lncRNA | 7SK RNA |
| ASHG19AP1B110408797V5 | 2.99e-09 | 92.2 | protein_coding | phosphoglycolate phosphatase [Source:HGNC Symbol;Acc:HGNC:8909] |
| ASHG19LNC1A100002945V5 | 2.99e-09 | 92.1 | lncRNA | novel transcript, antisense to ZNF260 |
| ASHG19LNC1A113684843V5 | 3.07e-09 | 91.8 | lncRNA | solute carrier family 7 member 7 [Source:HGNC Symbol;Acc:HGNC:11065] |
| ASHG19LNC1A104055872V5 | 3.08e-09 | 91.8 | lncRNA | novel transcript |
| ASHGV40004359V5 | 3.14e-09 | 91.5 | lncRNA |  |
