# Supplementary material for: Understanding Gene Expression and Transcriptome Profiling of COVID-19: An Initiative Towards the Mapping of Protective Immunity Genes Against SARS-CoV-2 Infection
Source: Front Immunol. 2021 Dec 15;12:724936. doi: 10.3389/fimmu.2021.724936 (PMC8714830; doi:10.3389/fimmu.2021.724936)
Supplement: Supplementary file 2 [file Table_2.docx]

**Table S2.** Top 250 differentially expressed genes (DEGs) and their sequence

| **ID** | **TRANSCRIPT_TYPE** | **SEQUENCE** |
| --- | --- | --- |
| ASHGV40004514V5 | lncRNA | GAAGGAAATGGGCAGGCCGGGGTGCTGTGGAACTCTCGCTGGCCCACCCTCCAGGCCTGG |
| ASHG19AP1B100079683V5 | protein_coding | AGGCGGCCGGGGGCATTAACTGACCTCTGAGTACAGCAATAAAATAACCTGGGGATCTTT |
| ASHG19LNC1A104444605V5 | lncRNA | AGGAGCCTCGATGTCTGCTCTCCAAGAGATGTCTGATCTGAAAGCATGTTGTGGTCCGCT |
| ASHG19LNC1A100061261V5 | lncRNA | CAAGGGAAGGTGGGGCATGTGTCTGGTTTCCACGTTTGTCAGGAGCTGTGAGCAGGAGCT |
| ASHG19AP1B143795176V5 | protein_coding | CTTGCTGGGGGGAGCCTGGGCAGGGCGATTCTGGAGGCCCCCTGGTGTGTGATGGTGCAC |
| ASHG19LNC1A100069148V5 | lncRNA | TCGGATGCTTTCCTAGATGTGGCTCTTCCTTTCCTAAGAGTGTAGATTGCTTCCTCCTCC |
| ASHG19LNC1A107586616V5 | lncRNA | TGGAAACCATTGGCAATAACACATCCTGGCATATGGCTCTGAGATGTATAAAGGCACAGT |
| ASHGV40012850V5 | lncRNA | GTATGTGGTCTGTGATGCTCCCAACTAAGCAGATGTCATCTAGCATTAGTTTTAAGGCGT |
| ASHG19AP1B130926225V5 | protein_coding | TGCAAGGGAGATGCCCAGCTCTCTCCTGTTTTTATAAGTCGCAGCTCCGCAAACTCACTG |
| ASHG19LNC1A100052836V5 | lncRNA | TAGTGGCCCCAAAACAGCTTCCTGACCTGCTTCTGTGGTCCCAGACCTCTTGCTGCTGAG |
| ASHG19AP1B130179979V5 | protein_coding | GGTCGTCCACCGCCGGATCTCGCTCCAGCGCCCGTGGTCCAGCGTGTAGGGAGCCGATCG |
| ASHGV40042878V5 | lncRNA | TAGTAGGAGCCTTGGAGGGTGGGTAGGTGCATGGAGGGTGACAGCAGCGCTGGTTCCGTT |
| ASHG19AP1B102939844V5 | protein_coding | ACGTAGGTGCGGGAACCTGGTTGAACCCCAAGCTGATAGGAAGATGTCTTCAGGAAATGC |
| ASHG19AP1B131783095V5 | protein_coding | GCCAGGGCTCCGCAGCGTTGCCGGTTGTATTCGCTGGATACCAGAGGGCGGAAGTGCAGC |
| ASHG19AP1B106458171V5 | protein_coding | GCTGGGATTACAGGGCACTGAATGTCAAAGTGAAGGAATTCAATGAAGCCCGGATCAAGG |
| ASHG19LNC1A105945162V5 | lncRNA | GGTTCCGTGGGGGTGCAGAGGTGGTTGCTGCTTCCAGTTTCCCTTCAGACTTAGCTGTGT |
| ASHG19LNC1A105982028V5 | lncRNA | GCCTGTGGGTGGATGTGCATGGTGTGGCCTGGAGAGAGAAGCTCCACGTGCAAACAGATA |
| ASHGV40002118V5 | lncRNA | TTACAAGTGCTGCCCTATCCATGGTGACTCCCCTTGGCCTCTAGGCTTGTCTGAGCAGAG |
| ASHG19LNC1A109596949V5 | lncRNA | CGGCGGGTGTTAGCAGATGTGGGATTTGAGACCCAAAGAAGTGAAGTAACCTGCCCAAGT |
| ASHG19LNC1A100048608V5 | lncRNA | TGAATTTCTGTTCCAGATGCCTCTCCACAGCATTCATCACACCTTCCTTCACACTTGAAG |
| ASHG19LNC1A101683633V5 | lncRNA | TCAATCCCAGCTCTTCCACTCATCAGCTTTGTTATCTTAGCCATTTTCTCAGGGCCAGGT |
| ASHG19AP1B101133016V5 | protein_coding | TCCTTCAAACGTCCCTGCCCCTTAGCACGATACAACCGCACCAGCTACTTCTACCCCACA |
| ASHG19AP1B127682210V5 | protein_coding | CGGTTCCGGTGGCGGCGATGGCTTCTCTTTCCACCTGGAGCAGCCCTGCAGAGCCCAGGG |
| ASHG19AP1B122326814V5 | protein_coding | TGGCTTCTCATTCCCCAGATGTGCGGGTCAGAGAGAATCAGATGGAGCCTGAGAGATGCA |
| ASHG19AP1B101848137V5 | protein_coding | ACGGCAGCCCACTGTCTCTACCAAGCCAAGAGATTCAAGGTGAGGGTAGGGGACCGGAAC |
| ASHG19AP1B100026912V5 | protein_coding | GACTGTAATATTTGCCTTCAAGATGGTTCAAACACAAAATATTAAATGAGCTGTTTCTTA |
| ASHG19AP1B100890801V5 | protein_coding | CCAGGTGGGGGTCACAGCAAGTGCAGATGGAGCTCAGATTCTTCGGTTAACTGTCACCCT |
| ASHG19AP1B136937830V5 | protein_coding | CGCGGGATGCTGGGCGGCGTCAGGTGAGCGGTGGTCGCTGGGCCTCAGGTAACCATGGAG |
| ASHG19LNC1A109148624V5 | lncRNA | GGACAGACCTGCTTCTGCTTTGTAATCATACGGACAGTTGTGCTTGTCAGAGTTAACGGT |
| ASHG19LNC1A100854911V5 | lncRNA | GGGCAGGCTCTCTCCAGTCTGAAAAGCCCACTTTCACTTGAGTTTCAGGTTACTGAGAGG |
| ASHG19LNC1A102103098V5 | lncRNA | TCTGCCCAGTGCTACTACATCTTTAATTGTTTTATTTTTCCCCAGGAGGAAGTAACTGTT |
| ASHGV40040540V5 | lncRNA | AATGTGATTTGTAAATGTATTAGTGATGAGGATAACAATAAAAATCAAATTTATAAAAAA |
| ASHG19LNC1A100084701V5 | lncRNA | AAAGAAATACAAAAGATGGTGTTAGGACCCTCTCCAAATTAAAATTCTGAATTTCCATGA |
| ASHG19LNC1A100048539V5 | lncRNA | AAGCTCTGTCAAAAGCTCTTGTTTCAAATCTATATTTGAATAGTAACATTTCAGGAAATG |
| ASHG19AP1B141920576V5 | protein_coding | CCTTTCACCAGGACCCGCCGGGCCCCGCCAGCAGGCGAAGCAGGGAGATGTCAGACTGCT |
| ASHG19LNC1A110630287V5 | lncRNA | AGCGGCCTGTGGGTGGATGTGCATGGTGTGGCCTGGAGAGAGTGCAGGACTCCCCCTCCT |
| ASHG19LNC1A100015590V5 | lncRNA | GGAAGGTGGTCTTGGGGTCTGAACAGTTCGTCCACTGCTGCTCAGGTAGGTGGTTTCACC |
| ASHG19LNC1A106045150V5 | lncRNA | TTGCCGCTAGAGGGTGACGCCTGGGTCGGAATCCAAGGATTTGTGAAGAGGTGACCCAAA |
| ASHGV40006178V5 | lncRNA | GAGTTTCCTACCCAGTATGTCCCAGTGCTCAAACTATTTTTTATTATCTTCAGAGTTTTT |
| ASHG19AP1B113236502V5 | protein_coding | GCAGCCCCCTCGGAAAACCACACTGAGAGTTGCATTAAAAACCAGCATGTAGGTATTTGT |
| ASHG19LNC1A102429018V5 | lncRNA | CCTGGGCCAGGTGGGGAAGGTTTGTTTGCAGAGCAAAGAAGTGAAGCCCTCCATTCTAGT |
| ASHG19LNC1A100092968V5 | lncRNA | GAAACCCCGCCCATCTTCCTGCTCATTGGCCGGTGCGGTTTACGTAAGAGGAGCCTGTTG |
| ASHGV40001413V5 | lncRNA | GAATAGCAGGGAAAGGCCATTGCTGCTCTGGAGCTGTGTGTGTGTGTGATGACTAAAGCA |
| ASHG19LNC1A106399525V5 | lncRNA | AGGGGGTAGAAAACATTTTGAAGATTTAGAAAGATCCTTCGAGTCGTATCTCAGAAAAGA |
| ASHG19LNC1A100063734V5 | lncRNA | GTTCTATGGACAATTGTCTCTCTGCGTAGATTAACCTTAAATAAACTTGTTGCGTTTTGA |
| ASHGV40010017V5 | lncRNA | GATACCCAGCTCTCTGCCTAAATCCTCTGTATGCAAATAAAGGTCTCTGCTTGTCTGTCT |
| ASHG19LNC1A100076220V5 | lncRNA | AATGAAGCATGGCCGTTTCTTCAAGAGAGACTTAAACTTCAAGCCTTTAGCCCTAGAGGG |
| ASHG19AP1B126722368V5 | protein_coding | AGATGCTGTTCTACTGGAGAAGTTCTAGATTCCCACTGTCCAATAGAAACACGTGAGCCA |
| ASHG19LNC1A100052346V5 | lncRNA | CGCAAACATTCTGTAAACACGGCGTAGAGTTCAGCCCTCTCCCAAGGTTCAGAAGGAGAG |
| ASHG19LNC1A104465952V5 | lncRNA | TGCCAGGTGGGGACTTAAAGCATACAGTGAAGTTGTTCTCTGAAGTTCCTCATCTGCCTT |
| ASHGV40059791V5 | lncRNA | TGTCCAAGTGTCCAGGTGTCAGGTGTCTGGGTGTCAGGTGTCTCGGTGTCCGGGTGTCCA |
| ASHG19AP1B116753425V5 | protein_coding | AGGCCAGCGCCGCGGTATCAGTAACATTCAAACATGTGACTATGGCCTTTACCCAGAAGG |
| ASHGV40053220V5 | lncRNA | CACAGGTAGAAAGTCACTGCCCTTTGGAGAATAGATGTTTACCTTTTCCAGAAACAGTCG |
| ASHG19AP1B139781923V5 | protein_coding | CCCGCTGCGCCGAGGGAGGGGAAACCCGAGGGGTTCCTTGGAGAAGGTGGTGCGTCCTGG |
| ASHG19LNC1A100020163V5 | lncRNA | CGACCTGCTTGGGGGTGTGATGCTTTCATGATGCTTCCTTTGATGTCGGGGGCCCTTGGT |
| ASHG19LNC1A104893279V5 | lncRNA | CACCAGCATGCAAATTTCTTCCAAGAGGGCGGTTCCGGCGGCAAGACGTAAGGAATGGCG |
| ASHG19AP1B124346093V5 | protein_coding | ACCAGGTGGGGGAGTTCTAGAAAGCAACGGGATGATAGATGCGGCAAGGACATACAGCAC |
| ASHG19AP1B100227479V5 | protein_coding | GTCCCTGCCGTTTTAGGACAATCAGGGCCCATCTTCTGCCAAGTGTCTGACCCCATGGGG |
| ASHGV40057731V5 | lncRNA | GAGGGTGCCAGCGGCCGCAGCTGAAGTTGGGCCGAGAGCCGGCGACGGCCCCGCGCCGGG |
| ASHG19LNC1A107625614V5 | lncRNA | ACCCCACTAATGAGAAGACACTTCCGACGGACTCAGACCCCAGCAATATCTCTCCCTGGC |
| ASHG19LNC1A101637126V5 | lncRNA | CCAAGGCTGGCGCAGTCGGTTTAATAATGATAGAATGAAGACCACAATAAAAGAGACCTC |
| ASHG19LNC1A110416853V5 | lncRNA | TGGACCTTGAGCATTCCTCAAGTAGGAAGATATCCCAGAGGAGACAGACATCGGATGGCT |
| ASHG19LNC1A100080479V5 | lncRNA | ATTCGTTCCGGTGATCCTCTCGGTCGCTCCCGTCGTACGAACTGCTACAGCTGCTCAAGC |
| ASHG19AP1B141555700V5 | protein_coding | AGGCAGCAGGCGCTTCCCCCGCGAAGGCCAACGGCCAGGAGAATGGCCACGTGAAAAGCA |
| ASHGV40026229V5 | lncRNA | TACACTGATCAGTTTTAAATGTCTTCTGGTGCAGAGCCCACAGAAACAGAGCTTCCCTGA |
| ASHG19LNC1A100076699V5 | lncRNA | TTAATCATTTCTGCCCTGTTACTCCCACCCCAGATCCAAGCGCCAGCCCAGTTCCGGTGG |
| ASHG19AP1B121660446V5 | protein_coding | CCAGCCAGGGAAGATGGTGCTCTGAATGTCACAGCAATGCCACCTGCACGGAGGATGAGG |
| ASHG19LNC1A100032732V5 | lncRNA | CCTTCGAATGAATAAATGATGAGAGAAGATACTTGTTGAAATCTTTTCAGAAATACCTAA |
| ERCC-00097_63 |  |  |
| ASHGV40050778V5 | lncRNA | TGAAAAACTCCTCCATGCCAAGCACTCGATTATGTCTGTCTTTGTACTCCACAGTGCCTA |
| ASHG19LNC1A101637125V5 | lncRNA | CAAGGCTGGCGCAGTCGGTTTAATAATGATAGAATGAAGACCACAATAAAAGAGACCTCT |
| ASHGV40059259V5 | lncRNA | AGGAGAGGGGTGTGGGCTGGGTGGGGTGTGAGCTGTGTGGTGTGTGAGCAGGAGAGGGGT |
| ASHG19AP1B100102680V5 | protein_coding | GGGTGGCGGGGAAGGGAGGAGGCTGAGAAGATGCAGATTAAATAAAGGTATGGAATGGAA |
| ASHG19AP1B140228294V5 | protein_coding | TCTCCGCCTGCAGGTGCAGACATCTGGAGGAGAGAGTCGGAGAGCAGAAACCACTTGGCT |
| ASHG19LNC1A102825717V5 | lncRNA | CCTGGGCCAGGTGGGGAAGGTTTGTTTGCAGAGCAAAGAAGTGAAGAGTCTTTTTGATTC |
| ASHG19AP1B127764973V5 | protein_coding | GGGACCAGCCCACCCGGACCTAACCCAGACCATCTCCCCCCGCCGGGCTACGATGACATG |
| ASHG19LNC1A109221690V5 | lncRNA | AATGCTCCAGCCCAAGTTACATATGTTTCTTGTCTTATGCATTTACAACAAGCCCTGGTC |
| ASHG19LNC1A108946762V5 | lncRNA | GCAGTTAGTCAGGTGCTGCTCTTTGTGGTGTGGTGGGCTCTGGTCTGTTCTGCTCGGTGC |
| ASHG19LNC1A103685577V5 | lncRNA | CACTTCCTTCTGTCAGCTCTCCTTCCAACTCACACTTGGGCTTGGATGGTCTCACTGGCA |
| ASHG19LNC1A100032333V5 | lncRNA | AACCCAGCTCTTTCAACTCCCAAGGTTACTTTGTCAATAAAGTGCGCCTTCTCAAGTCTT |
| ASHG19LNC1A100076703V5 | lncRNA | CTTTATTCTCACTCTAAATGAAATGAAAGAGCTAAATAAACAGTAACTGTCTGAAGGAAA |
| ASHG19AP1B105392872V5 | protein_coding | GCGGGTCATTACGCTGAGAAAGTCCCTCCTGGTGCATCACAGTCGCCAAGCCGTGGAGAA |
| ASHG19AP1B108131779V5 | protein_coding | GCCAAGAGTATGAGGAGCTGGTGTCTGTGTCAGATTTGTAGCTGCGGGTCGGATTATTGT |
| ASHG19AP1B109420977V5 | protein_coding | TCCTCTTATCACAGGTGAGGAAACTGAGGCCGGGGGTTCAGAGCAAGAGACAGAGGTTCT |
| ASHG19AP1B102211524V5 | protein_coding | TCAACAATTTACCCGATATCAGGTTCTTCTTTAGACTGGGCTTATGACCTGGGCATCAAA |
| ASHGV40059449V5 | lncRNA | GCTGCATTCAAGAATGTGAGCTCTTTTCTGTGTCTGTGGAGTAAAATGAGGAAGTTATCA |
| ASHGV40001229V5 | lncRNA | TTCTCCTTTGGAAGGATCTCTCTGGCCCATCTGGGGGAGGGCAGCCCAGAGACACGTGTG |
| ASHG19AP1B100262006V5 | protein_coding | TCTTCCACATCCTGCTGCTCTCGGCGCCCCCGGTGCTGTTCGTCGTCTACTCCATGCACC |
| ASHG19AP1B100226035V5 | protein_coding | CCGACCCAGCCCAGTGCCACCTCTATCTGTGTACATACTCCCTTCTTCCACCCAGATGGG |
| ASHG19AP1B116099560V5 | protein_coding | TGGCATGCAAACGTTAGAACCAAGTGTTGTCTGTGTTGGTGGAGATGGATCTGCTAGCGA |
| ASHG19AP1B119162740V5 | protein_coding | AGCCAGAGCCGCCTGCCATGCCCCAGCCAGTCCCCACAGCATAACAGTTTCTTACTTTTG |
| ASHG19LNC1A109158522V5 | lncRNA | TCAAAAAGACCAAGACTTGATCCCTTGCCCACAAAGATACAAGGCTGAGGACAAAGCCAT |
| ASHG19LNC1A100454332V5 | lncRNA | TAAATCCTAAGTGGCGTCGGACCCCTGTCAGAGAGTAAATCTCAAGTAAGGTACCTGCCA |
| ASHGV40001197V5 | lncRNA | AGCAGCTCGATGCTCTGGTTACCAGATTCGCGTTTACTTTGGAAAAATCTAAGATCGCCA |
| ASHG19LNC1A100073073V5 | lncRNA | CTAGTCACCGTTTATTTAAATGACCTGCTTCTAAGAACCAAGTGAGTTTTGTATTCTGGC |
| ASHG19LNC1A102092106V5 | lncRNA | TGCCAGGTGGGGACTTAAAGTTTCAGTTACTCATTGTTGGGGCTCAGAAACCAATACCCC |
| ASHG19LNC1A100011085V5 | lncRNA | TGTCCTGTGACGTAGCCCTTTTCAAAACCACCTCTCCCAAGTGCTGCACCACTCCTCCTC |
| ASHG19LNC1A111689598V5 | lncRNA | GCCCTCGCTGGTTCCCCCCAACTCAGGTGGTGTTTGAGAAGGGCGGAGTGTACCTGCACA |
| ASHG19LNC1A107535210V5 | lncRNA | TGGTCCTGGACTCTTTTTGGTTGGTCTGCCTTATTATTTTCGTCAGTGTTGTGCAGGAGA |
| ASSPINKEIN100003643 |  |  |
| ASHG19AP1B104673574V5 | protein_coding | GGACGTCCCTGCCCTCTGCCTTGCCTCGTATTGTGGTTCACTAGTAAGTGCCTGCTTCCC |
| ASHG19LNC1A100093030V5 | lncRNA | CCATAGACAGTATGAATGGGTGGGTCTCTGTTCCAATAAAACCTTATCCTAAACACTGAA |
| ASHG19AP1B115327726V5 | protein_coding | CTTCCAAATCGTGGATCCCAAACACCAAGAACATCTGGTGCTCCAGTTCGGCCGAGTGGG |
| ASHGV40051007V5 | lncRNA | AAGTTACCCACATTTCTGCCCAGCTGACTACAAATTCTGGTTTCCATGACCCCCTCTCAG |
| ASHGV40052827V5 | lncRNA | TATTCAGTCAACAGAAGCATTGCCCTCACTTTATGGGTCACACATCACATGGGACTGTGT |
| ASHGV40033809V5 | lncRNA | AGAACGAGCCATCTCCTGGCTGGTTTAGCAGTGTGGGATTGTGTGGTGTTTCTATTAGTC |
| ASHG19LNC1A104002307V5 | lncRNA | AGTTTCATCACCTCTCTACAAGTTTCACGCTGTTTAAGTCATGAACCTACAAAAGCCTGT |
| ASHG19AP1B118570399V5 | protein_coding | ACCTTGAGAAATGGGTGTCCTGTGTCTCTTTATTCTTGGGTGGGTAGGTGGGTCAAGCAT |
| ASHG19AP1B102926930V5 | protein_coding | GCCAACCTACTAACAGGTGGGTGGGTATGGTGTGTGGTTTCACTCAGTTCTTCTCATGGG |
| ASHGV40040841V5 | lncRNA | ATATGCTTGCCCTGTGGCTGTGTCTGGCTATACAATTCCTGGAACAACTCAGTCCTGGAG |
| ASHG19LNC1A100048727V5 | lncRNA | CTTCTATTGGTATTAATTCGGGGCTCTGTAGTCCTTTCTCTCAATTTTCTTTTAAATACA |
| ASHG19AP1B100010099V5 | protein_coding | TCTTCCTTTTTCAATCTGGAAAACTGTCTGACCATGAGGAGAAGCCCCCTCAGCAGTGAG |
| ASHGV40016060V5 | lncRNA | TCATTTTTGTAACCCCAGGATGCCAAGCACAACACCTGTCACAGAAATTCAGCAGTTTTG |
| ASHG19AP1B100143862V5 | protein_coding | CTAAATGAAATTGAGAACATGCTTTGTTTTGCCTGTCAAGGTAATGACTTTAGAAAATAA |
| ASHG19AP1B127343616V5 | protein_coding | CTGCTCAGCTGGTGGGTATGGGTCCCCTATCTTTCTAGAACCAGTATGTGGCATTCCTGT |
| ASHG19AP1B141086300V5 | protein_coding | AAGCCTTCTGCCTGTGATGGGATGATTGCTGAGTGTCCTGGGGCCCCAGCAGGCCTGGCC |
| ASHG19AP1B133251296V5 | protein_coding | TACGCCTCGGGGATCAGAGAGAAGCGAGGTTCTCGTTCTGAGGGACAGGCTTGAGATCGG |
| ASHG19LNC1A107787072V5 | lncRNA | AGCTGAAGTATTATGAACTCCAAATAATGCTTTGAGGACCTCCAAAGGGATTAGTAGTTT |
| ASHG19LNC1A100052843V5 | lncRNA | GAACATCCCTGCCCGTGTCTCAGCGCAAATACACACCAATAAATCCATGTTTCCAACTCC |
| ASHG19AP1B116251415V5 | protein_coding | GGGTGGTTCCGGTGTGAGCTCATCTTGGTGTGTTGTGGGGTGTAAGGAGCAGTGTGAGGG |
| ASHGV40028330V5 | lncRNA | TTGCATAACAGAAGATGTATCAGGTCTTTGTCCTGGATCCTCGGAGGAAGCTTCTAAACC |
| ASHG19LNC1A108788791V5 | lncRNA | CTCTGAAGGCCTTTCTGCCCAGTCTGCCCTCTTATTCCTCCTGCAGGTCACGACCCCCAG |
| ASHG19AP1B100207377V5 | protein_coding | GCTTCCTTGAACTTTGTGTTAAAAACAGTTCTGCTTCTGAAAATAAAGTTTTTAATCAGA |
| ASHG19LNC1A109555842V5 | lncRNA | AGTCGGCTCTCAGTCCCAGAAGTGTTTGGATTGATGAAGTCTGACCCAACTTGCATGGCT |
| ASHG19LNC1A100034530V5 | lncRNA | GAGGGGCTACCGTTCTGCCTCCGACAGGTTGTGTGTGGGTTTACTTGGAGGTGCTTTGCC |
| ASHGV40030424V5 | lncRNA | CTTGTCCCTGCCCCGCGTTGCCTTTTAAATTTTAGCTCATTCCGAGACACCTGCCGTCAG |
| ASHGV40034868V5 | lncRNA | AAGGAAGGCATTACCAGCAATAAGAAGTGATAGCTGCCACGGGAAGCAAATGCAGAAACA |
| ASHG19AP1B105033128V5 | protein_coding | CCAGGAGAGTGGTGTTTGGGTGTGGAAGGGGTAAGGCTCTTTCTCTGGGGCGAAGTGGGA |
| ASHG19LNC1A109552992V5 | lncRNA | TTTCTTCAGTCACATCTGAATAAATCCCTTAGAGTGTCTGTGTGCTATGAAGATTGCACA |
| ASHG19LNC1A100072148V5 | lncRNA | GTTGTGGAGATGGATGGTGGGGATGGTTACACAACAACGTGAATATGCTTAAAGCCACGT |
| ASHG19AP1B102268874V5 | protein_coding | AGACCATCTCCAGAAATTGGATGAGCAAAAGAAATGGTTAGATGAAGAAGTAGAGAAAGT |
| ASHG19LNC1A100004110V5 | lncRNA | TGTGTAGGCCTAGGCTAATGTGTGTTTGTGTCTTAGTTTTTAACACAAAGTTTAACAAGT |
| ASHG19LNC1A100057076V5 | lncRNA | GTTGCTGTGGGGCTTGGGGATATGTTGGATTCTCACTGGCCCGGAACTTTGCTCCCCGAG |
| ASHG19LNC1A110290204V5 | lncRNA | GGGCTGTGGGGTTTGGGCTGGAGTGCGGTGGTGTGACCTCGGCTCACTGCATCCTCCGCC |
| ASHGV40029603V5 | lncRNA | GGTATCTCAGTACAGCTTCTGGAACAAAGTTCTCTGCCCTTTCTCTTATTGTTATTTTTT |
| ASHG19LNC1ABL100000566V5 | lncRNA | CGAAAAGTGTGTGCTGACAACTCTGGAATACACAGTAGGCACCCAATACATACAAGGGAG |
| ASHG19AP1B108733567V5 | protein_coding | TGTATGACGTTGCTGATCACTGGAGATTCCATCGTTAGTGCTGAGGCAGTATGGGATCAC |
| ASHG19LNC1A100887318V5 | lncRNA | TCCCCGGGGAGATTCGTTCTCATTTTTCTACTGCTCGTGATGTTCAGCCCACAAGAGCTT |
| ASHG19AP1B106402435V5 | protein_coding | GCTCCGCAGGCTGCCGGCTCCACCCCTCAGATTCATTTGGATTCAAGGTTGGCTCTCAAC |
| ASHG19LNC1A100089080V5 | lncRNA | GTTTCACATATAAATTTGGATGCCTTGCTATTTGGGGAAAATAAATGTGAGCTCTGGCAA |
| ASHG19LNC1A110321113V5 | lncRNA | GGGTCGGTCCTCTGAAGAGGCTGGGGCGTCATCGGGGCCGGTTAGAAGCTCTGCTCCCCG |
| ASSPINKEIN100008173 |  |  |
| ASHG19AP1B100077547V5 | protein_coding | CTAGAGCAGGCCAGGTCATCTTTGGGTGGTGGAGTGCAAAGGAGGCGACCTGCAACAGAG |
| ASHG19AP1B135988649V5 | protein_coding | CAGGACTGGTGCCAGCTCCGCGTTGTTCCGCGAGAAAGCGAGAGGCCGAGCCCGGGCTGG |
| ASHG19LNC1A100093161V5 | lncRNA | ATTCCGGAAATATTGGAAATTAAGCAACATAATTCCAAATCTCTAGGTCAAAGAAGAGAT |
| ASHG19LNC1A100061364V5 | lncRNA | CAAGATGTGGAGGTGGAAGACAGTGACACTGATGATCCTGACTCTCTGTAAGCCTAGGCT |
| ASHG19LNC1A110168759V5 | lncRNA | CTCTCGCTGGCCCTGCTCGCGGATCCCGAGTAGAGAACGCAAGCACCCACGCCCGCCTGC |
| ASHG19AP1B100095122V5 | protein_coding | GGATAGCATTAGGACAAATATCTAATGTAAATGATGAGTTAATGGATGCAGCAAATCAAT |
| ASHG19LNC1A108801755V5 | lncRNA | TCCTGCTGCCCGCCACCATCACCACTGCAACTGGGCCCAGCAAAATCATAGAAGGAAACT |
| ASHG19LNC1A100044793V5 | lncRNA | AAGGTATTATCAAGAAAGTGAAAATACAATCCACAGATATTGCAAATCGTATCTCTGGAG |
| ASHG19LNC1A112100487V5 | lncRNA | CCCAGCGCCGGGCCAATGTCCTATCTCAGGGTTTCCCACGTCGCTCGCTCCTTGACGGCT |
| ASHG19LNC1A100835893V5 | lncRNA | AGAGTGGTGGAGTGAGTATTGTCATCTGCATGAGCAAATCTGGTTCATCACATCTTTGCA |
| ASHG19AP1B127249610V5 | protein_coding | CCGGGGTGGTTGGCATTGTGTCCTGGAAGCGGCCATTCACTCTAGTGATCTCCTTCTTCT |
| ASHG19LNC1A107590865V5 | lncRNA | ACAATGGTGCCCCCTCGTGACAGAAGCTTGAACTACAGCTTGAACATCGTGTGACATGAT |
| ASHG19AP1B102078045V5 | protein_coding | AGCAGCAGGTTCTCCAGGTGAAAATAAGTCCCCTCCTCGCCCATGTGGCTTGAATCACTC |
| ASHG19LNC1A100076086V5 | lncRNA | GATGGTTATGGGGACCTTGCTGATGTGTTTGAACTTTATGAGTGTGAGTCCTGATCCAGG |
| ASHG19AP1B100171364V5 | protein_coding | CATGTAACTGTCTGTGTCTTGCTTTTTGTATTTTTATGAAGTCTTTGGAGAAATAAAAGT |
| ASHG19LNC1A106002067V5 | lncRNA | CGCTCTGTGTCCTGTGAGAAAGCGGATGATTCCAATGTACAGCCAATGATGAGCAGCTGC |
| ASHG19AP1B118160980V5 | protein_coding | CTTCCATCTTCAGGACTCGTGCCTTCTGCTGTTCTCATTGTGGCCAAGAAATGTCCCAGC |
| ASHG19LNC1A100012534V5 | lncRNA | GAAGCACAAGTACATCTGGTAGATGCCCATCTCCCCCACCGCCTGGAACGCCTCCTCCAC |
| ASHG19AP1B100044033V5 | protein_coding | TAGGGAGTCTCGCGTCCACACCGCCCCTTCCCTTTGTAGCAAGCTGACAAGGAGACACTC |
| ASHG19LNC1A101254421V5 | lncRNA | TCACTTACAGACTGCCCCTGTTTCAGCCTCCAGGAGAGTCTGTGACAAGGAGTGGGCTCC |
| ASHG19AP1B101393687V5 | protein_coding | AGTGTCAAGCTGTACCGCCACTCTGTGCTGGAAGTGCTGACAGCGGCTGTGGAGTATGGG |
| ASHG19LNC1A106434347V5 | lncRNA | AGGGGATCAACGGGAAGGATGGAGAGAACAGCTTGTGTGCCAGGTGGGGAGACGGACATG |
| ASHG19LNC1A112379221V5 | lncRNA | CGCTGCCAAGCTCTGGGGGTCATCACAGAGAAGGAGACCCAGGTGGCATCACCTGGAGCT |
| ASHG19AP1B132472269V5 | protein_coding | TTCCCAACTTGATAAGGCGACCGTGGCAAAATTGGGGCCTCTCCCTCGTGTTCTTGCTGA |
| ASHG19LNC1A100057683V5 | lncRNA | CCTGGTTCCAGCTCTTCCCCAGCCTGGCTGTGGTGCCTTGTTCAGGGTAAATACTTCCCT |
| ASHGV40051914V5 | lncRNA | TTTCTGGAGTGATTTTCTCTTAGATTTCTGAATGATTAGCATACCTTGACATGGTAAACC |
| ASHG19LNC1A100075651V5 | lncRNA | TGTTCTGTGTCGTGTCCAACACACTTTGTCATTTTCTTTCTAATCCTTGTGATTATTATT |
| ASHG19AP1B104650775V5 | protein_coding | TCTGTTTCCAGTCTGTGGTGATGCTGTCTGTCTGTCTGAGTCTCGTGGCCGCCCCTGGAC |
| ASHG19LNC1A110644885V5 | lncRNA | CAGGCGCCAGGTGGGGTGAGTGAGGCTGTCCATCGAGGGCTCTTGGGGGGGTGTGGGCTC |
| ASHG19LNC1A101660177V5 | lncRNA | TTATTCTCATGCAGCTTCCTGTGTCCCTGTCCTTTGGAAAATGCTGCCTCCTGGTCTGCT |
| ASHG19AP1B124077767V5 | protein_coding | AGCACTGGAAAATAGTTTGAAATTAAAAGGGAATAATAAGTCACAAAGGGAGGAGCTGGA |
| ASHG19AP1B117393755V5 | protein_coding | GGCTGTGGCTGTGAGTATAAAATCTTTTTAAGATCTCAGAAAGGCCTGATGTGGCACCCC |
| ASHGV40004388V5 | lncRNA | GATCGAAGCAGATGCAGTCTTTCACATGAATATTTGAATCAGGCACAGGATGCAGGTGAT |
| ASHG19AP1B120812793V5 | protein_coding | GCACCTGCTCCAGGCTATAATGGTAGAGAAGGGCCCCCTCATATACCTCGGTCTCGCAGG |
| ASHG19AP1B110829606V5 | protein_coding | CGCAGCGCCCCGCGTGCCGATCTTTTCCTAATTCAGCAGCGATTTAACCAAGAGCCTGGA |
| ASHG19LNC1A100028094V5 | lncRNA | TTGGTGGGGTGTGGTGAGATACTTGCATTGTATTTTCAATTAGCAGCAACATTCTATGGT |
| ASHG19LNC1A106532476V5 | lncRNA | TGGTGTATCAGCATCTTTTATACCTGAGTAAAGCAGAAAGGTTTAAGTTTACCACTTTTT |
| ASHG19AP1B100140590V5 | protein_coding | GCTCTGAGAAGTTGGAAGATCTGCTGTGCTTCCAGACACCCTCTTACCCTCTAGGAGGCA |
| ASHG19AP1B104000725V5 | protein_coding | GTGCATGGCTGTGAACTGGAATGTGTGACTGTGACCTTATGGCTGCCGCACGCCTCTGCC |
| ASHGV40023539V5 | lncRNA | CTGCCTCAGTGCTTGATTTTTAACGGTTTTGAAGAGAATAGTTACATTTCTTCTCCTAGT |
| ASHG19LNC1A102866939V5 | lncRNA | CCGGGCCTCAGAACCAGGAAGATCCGGATAAAGACACTGTCTGCACTCTACTCAGATGCC |
| ASHG19LNC1A106013140V5 | lncRNA | TTCCCAGCTTCTCCGTGCTGATACCCCATGGTTCTCCTACCACCAGATGCTACTGAGGCT |
| ASHGV40048341V5 | lncRNA | GCCTATTGTTGAAGGTCGGCTCTTTAATGTGCAAATTAAGGTGTTTTATTTCCTTCATAT |
| ASHG19AP1B133038450V5 | protein_coding | GGCTGAACCCCGGGTCTGCGCCTTTCCCCCAGCCTGGAGACGATGCCAAAGCTGCAGGGC |
| ASHGV40021624V5 | lncRNA | ATTAGTTTAGCCAAAGGATAAGTGTCCCCTATGGGGATGGTCCACTCTCACTCTTTCTCT |
| ASHG19LNC1A102085612V5 | lncRNA | TGATGCCAGTTAATTACTTTGAATAGAAGACTCACTGGTGGCACCTGCAGCTGCGGCGTT |
| ASHG19LNC1A102428297V5 | lncRNA | TGCATTGGAACATGACATGAGATTAAGTGTATAAAGCCAAATGTCCCATTTAAAGTTTGA |
| ASHG19AP1B101019195V5 | protein_coding | GGCGCTGCGAGCAGAACCCAGTGACTTGATTGTAAAAATTTACAGAGCGGAATCATATGC |
| ASHG19LNC1A100088080V5 | lncRNA | CACCAGGTGCAATCTCCAACATGTGCGGGCTTTGGGAAAACACAATCGGGCAAGTGAAGT |
| ASHG19LNC1A100482211V5 | lncRNA | ACTTGTTTGTTGAATAGATACATGAAAAGAAAGAAGGAGAGAAGGAAGAAAGGAGGGCGG |
| ASHG19AP1B100197615V5 | protein_coding | GGTCAGGCATCTTCCCTGCCGTCGTTTCTGGGAGGTTGAAAAATTGATCCAGAAAGACCT |
| ASHG19LNC1A100072825V5 | lncRNA | TGTGTTAGTCTGGAAGCACCAAGCACCGCATACCAAAATACCTCGAGGCAACCGTATGGC |
| ASHG19LNC1A106475763V5 | lncRNA | GCTGGAGCGTCGGGCCAGGAAGACGGAGAACTGGATTTGCTGCCAAACTCATTGAGGGTG |
| ASHG19LNC1A105245011V5 | lncRNA | AATTTGTGTGTAATTATAATGTTCTATGTGTGGTGTTATCAAAAGAATCACTGTGTCTCT |
| ASHG19LNC1A100012092V5 | lncRNA | GTGTCTAGGTTTGCCTTTGTCTTTTTGTTGTCAGAATTTCATTAAACTAATTATTCATTC |
| ASHG19AP1B143197067V5 | protein_coding | CCTCTGGCCCATTCCCTGGGGAGTGGCTGGAAGCGAGGAACGCAGTGGATGCCTTTCGGG |
| ASHG19LNC1A102812538V5 | lncRNA | TCAAGTGATCCTCACACTTCAGCCTCCTGAGCAACTGAAACTACAGTGAATAAGAATGGT |
| ASHG19LNC1ABL100000870V5 | lncRNA | AACTTTTCTGTTTGAATGTTTGATAATAAAATATTGAATAAAAATCTTTTTGCTAAAGAT |
| ASHG19AP1B111702966V5 | protein_coding | CAAGATGCCTGGGTGAGTCATTGGTAGGGAGGCGAGTGCTGGCGTGACGAGATGCTACGG |
| ASHG19LNC1A100084341V5 | lncRNA | GAGAATCCTGACCTTATGCTGGCCTAGGCTAATGTGTGTGTTTGTGTCTTAGTTTTTAAC |
| ASHGV40019012V5 | lncRNA | CTGGGGCCTGGGGGATGGAATAGGAAGGAGATGTATGCTCTTTACTCTACATGATTTTTT |
| ASHG19AP1B108818018V5 | protein_coding | GGAAACTGTTTTGGATGATCTTGACCCCGAGAATGCCCTTCTGCCTGCAGGGTTCCGGCA |
| ASHG19LNC1A106039798V5 | lncRNA | AGGGTGGAGGTGTGGATGCTTAACCCAGTCTTTTTGCACCCATAGCCTCTTCAAGTTCCT |
| ASHG19LNC1ABL100000681V5 | lncRNA | GGGTGGGGACATGCAGCAGATGGGCCGATTGTAAAGGAATAAATATAACATTTATTTTCA |
| ASHG19AP1B107974207V5 | protein_coding | TCAGGACAGCAGATTAGAGGCAGGAGGCAATGACAATAAAATAACGATAAAATCCTGAGA |
| ASHGV40024194V5 | lncRNA | TCCAGGTATCTGGGTGTATGGGTGTCTGAGTGTCAGGTGTCTGAGTGTCCAGGTGTCCAG |
| ASHG19AP1B102930566V5 | protein_coding | GGCCGCACCGTGGACTTGTTCTTAATCGAGGGGGTGAGTGAGGGGTCTGTTTCAGTAGGT |
| ASHG19LNC1A100060784V5 | lncRNA | GCAAGAATTTGTAGACTGCTCTAAGCATTTTAAAGTTTATCTTAAAAGCAGCTTGATAAA |
| ASHG19AP1B124609586V5 | protein_coding | ACTCATGACCAGCCAGATGTCTTAAGGAATGCTTTTGAAAAGAACAGAGGCGTACAACAT |
| ASHG19AP1B101292487V5 | protein_coding | TGGCTTCTCAATCCAGGTCCCTGCCCTCCCTGTTTTGAGGTTTAGACAGGATGAGAGTCC |
| ASHGV40037100V5 | lncRNA | TATGTCCACTCTGGATGTCCACAGTGACTCGGGCTCCTCAGCAGCCCACGTCCTCCTCAC |
| ASHG19LNC1A109427515V5 | lncRNA | TCTGACTGCTACCCCACTATTTTGAAGCATACATGGGAAGCTGGTTCCTGGACCCCCGCA |
| ASHG19AP1B100785967V5 | protein_coding | AGGCCGACCTGGGGAAAGACTGATTTTGAGGTTTTAATAGTTTTCAGATGCTTCAAGTGT |
| ASHG19AP1B142926223V5 | protein_coding | GTTTGTGTCTGGAGCCGTAGCGGCAAGTGGGCTTGCGGCTAAGGGATTTTCCTGGGATGA |
| ASHG19LNC1A108832336V5 | lncRNA | TGATGTACAGGGATCTGTGGAACTTTGAACTTGAGAGTGATGATTTATGGTATCTGGTGG |
| ASHG19AP1B103461095V5 | protein_coding | TGTGACTGTGTCTGTGACAAGCAGCACCATTTCATCAAATGTGGCATCCAAGGCTGCCTT |
| ASHGV40029368V5 | lncRNA | AAAGAGCCAGGCCCTCGCTGCCAAGGAGCTTTCCCGTAACACCGATGCATCAAACAAAAG |
| ASHG19LNC1A104876954V5 | lncRNA | GGCCTCTCCTAAACCAAACAGGACGCCACGGGCAAGTTGGAGCAAAGTCCTGGCAAACCT |
| ASHG19LNC1A109294555V5 | lncRNA | ACCAGCTACGAAAAGAATATAGCTGCTCTTGAATGTGAGGTTTGTTGGTATAAAAAGTGT |
| ASHG19LNC1A100028099V5 | lncRNA | GTGAGAGTTTTCTGCCCTAGTGCCACTGCTTTAAGAACTACAGCTTGGTCATCTCTAGAA |
| ASHGV40051026V5 | lncRNA | TCTGCCCCAGTTATCCATTTTACTGGATAGTCTTGGTATCCAGATGATTTAGGGAAAAAA |
| ASHG19LNC1ABL100000390V5 | lncRNA | CAGCTCTGCAGGCATTTATTGGCAGGCTACACCTAAGACTTTCTCTGCAGAAAAATGTTC |
| ASHG19LNC1A108131956V5 | lncRNA | GCAGAAGTGTGTCTGTGCATCTGTGTCTCTGTGCAGGAGGCCTGTGCAACCTGTGCCCAG |
| ASHG19AP1B100219166V5 | protein_coding | CAAAGCTGTGTTCTGTTGGCTGGTGGAGGACCTCACTACCACAAATAACATGCCCAGGGC |
| ASHG19AP1B134807541V5 | protein_coding | TCTCATGCCCTGTATGCCCTGTGCTCTTCCCACAGGTGGCCTTTTGCCCCACCCCCAGCA |
| ASHG19AP1B124487458V5 | protein_coding | TGCAATGAAACTTTTCCTGAAAAATTAGAGGACCCACAAGGAAATCTTGGAAGTTATGCT |
| ASHG19AP1B116871577V5 | protein_coding | ACCAAGAAGTTCCTGCCTTTTGTCTCTGAGCCTGATGTGTGTAGGGGTGATGGAAAGGCT |
| ASHGV40050040V5 | lncRNA | TCTTACTTTCGTTACTCATCTTATCAAAGTAGGATAACTTGGAGGCAGCGTAGATGGCGC |
| ASHG19LNC1A100043936V5 | lncRNA | TATGTGGTCTTCGACGAATACTTCCACCCCTGTGTGGTGCAGTTTGCTCATCTGCAAAAT |
| ASHG19LNC1A100024725V5 | lncRNA | AAGAAAAAGTTAATTTTTCAACAAGTTCAGGCTCTTGCAGAAGACCTTGAGCAGCATGGG |
| ASHG19LNC1A109170827V5 | lncRNA | GGCCTGTGCCTGTGGTAACTGTCTATGAGCCAGGTATATCTGAAGCATATTTGACAACAG |
| ASHG19AP1B127674344V5 | protein_coding | CTGTGGTAGGAAGGGAGGTCCGCTCGGCCGGGTGCGCCGCCCCAGTGCTCTGTGGGATAC |
| ASHG19AP1B112128398V5 | protein_coding | GCGAGAGTCGGAGATATGACTGACTGATTCCTTCTCTGGAATAGTTACTTTACAACACGG |
| ASHG19LNC1ABL100000341V5 | lncRNA | GGGTAGGTCTGGGTACAAAGCACTGGGAATACAACCTTGAACAAGACAAGAATCCTGGCC |
| ASHG19AP1B114603136V5 | protein_coding | GCTGGCGCTCTATGAAACCTGCGAAAAGTACGGACTTGGCTCACAAAATATCATCGACTT |
| ASHG19LNC1A107597508V5 | lncRNA | AGGGAGACTGACACATTGATCACTTTCTCAACCTTTGATCTCTTGAGAGGATGGTTCACA |
| ASHG19LNC1A106435265V5 | lncRNA | CCGGGGGAGGCTGCAGGTTCACGTCGGTGTCCGGTGATAAAGAAGACACGGATACAGCAA |
| ASHG19LNC1A100025009V5 | lncRNA | TGTGTGTGGGGAGGGGTGCAGGAGGTGGGTGGAGTTAATGGAGTAGTGGTTGTATGGTAC |
| ASHGV40027552V5 | lncRNA | AGATTTAAAGACCCACATTGTTAACAATGGTGTTTTAGGTGTGTGGCGTATTAGAGGAGT |
| ASHG19LNC1A100048535V5 | lncRNA | CTCAAAGTACTCTGCTAAGGGTAATTAAAAGGAATAGGATGTTATGAATTAGATGAAAAA |
| ASHG19AP1B100132859V5 | protein_coding | TATCCTAGTACCTGTGTGCTTATGTCCGTGTGTGTCCGTGTGGCCTGGAGGAGCCCGAGG |
| ASHG19AP1B140078097V5 | protein_coding | TCGCAGCTCCCGACTGAGCTGCGCCTGCGCAACTCATTGGCGCCAAGATGGCGATGGAGA |
| ASHG19LNC1A100032095V5 | lncRNA | AAGGTAGGGTAGTCAAGATTCCAGACACATCCAAATGAGGCGCTGCATGTGGCAATCTGC |
| ASHG19AP1B110408797V5 | protein_coding | TAGCTGTGCTCTGTGTCGTAGGCAAAATTCCACTGAAGGAGCCTCTGTGCCCTGCAGCCG |
| ASHG19LNC1A100002945V5 | lncRNA | GTTTACAGAGGCCACTTAAAATCCTTACGTTAACCCTAGGCATACTGTGGTCATCCTTTT |
| ASHG19LNC1A113684843V5 | lncRNA | ACGCTCCGGGGACTCCGAGTCAACAGCTGTTGCAGCATGAGCGATACGCTTGGTTCTCCT |
| ASHG19LNC1A104055872V5 | lncRNA | TGACACCCTGCCCAGTTGTCCCCTTGGAACCTGTAAACACTTTGGCATAAAGATCTGACA |
| ASHGV40004359V5 | lncRNA | AAGAATGGCAGCAGGGTCTGTGTGTGGTGGGAAGCAAACTGGCCTGAGG |
