# Supplementary material for: Understanding Gene Expression and Transcriptome Profiling of COVID-19: An Initiative Towards the Mapping of Protective Immunity Genes Against SARS-CoV-2 Infection
Source: Front Immunol. 2021 Dec 15;12:724936. doi: 10.3389/fimmu.2021.724936 (PMC8714830; doi:10.3389/fimmu.2021.724936)
Supplement: Supplementary file 3 [file Table_3.docx]

**Table S3.**Top 250 differentially expressed genes (DEGs) and accession number and chromosome

| **ID** | **TRANSCRIPT_TYPE** | **ACCESSION NO** | **CHROMOSEME** |
| --- | --- | --- | --- |
| ASHGV40004514V5 | lncRNA | NR_110947 | chr16 |
| ASHG19AP1B100079683V5 | protein_coding | ENST00000255039 | chr1 |
| ASHG19LNC1A104444605V5 | lncRNA | NR_148363 | chr14 |
| ASHG19LNC1A100061261V5 | lncRNA | FTMT21000006986 | chr3 |
| ASHG19AP1B143795176V5 | protein_coding | ENST00000320838 | chr19 |
| ASHG19LNC1A100069148V5 | lncRNA | ENCT00000222744 | chr2 |
| ASHG19LNC1A107586616V5 | lncRNA | ENST00000608176 | chrX |
| ASHGV40012850V5 | lncRNA | T097905 | chr13 |
| ASHG19AP1B130926225V5 | protein_coding | NM_001318905 | chr10 |
| ASHG19LNC1A100052836V5 | lncRNA | ENST00000649453 | chr6 |
| ASHG19AP1B130179979V5 | protein_coding | ENST00000342782 | chrX |
| ASHGV40042878V5 | lncRNA | ENST00000412295 | chr5 |
| ASHG19AP1B102939844V5 | protein_coding | ENST00000262746 | chr1 |
| ASHG19AP1B131783095V5 | protein_coding | ENST00000369076 | chr6 |
| ASHG19AP1B106458171V5 | protein_coding | ENST00000602198 | chr19 |
| ASHG19LNC1A105945162V5 | lncRNA | ENST00000562514 | chr16 |
| ASHG19LNC1A105982028V5 | lncRNA | ENST00000419183 | chr3 |
| ASHGV40002118V5 | lncRNA | ENST00000555460 | chr14 |
| ASHG19LNC1A109596949V5 | lncRNA | ENST00000509458 | chr5 |
| ASHG19LNC1A100048608V5 | lncRNA | ENST00000619099 | chr17 |
| ASHG19LNC1A101683633V5 | lncRNA | ENST00000558818 | chr15 |
| ASHG19AP1B101133016V5 | protein_coding | ENST00000536005 | chr16 |
| ASHG19AP1B127682210V5 | protein_coding | ENST00000337665 | chr19 |
| ASHG19AP1B122326814V5 | protein_coding | ENST00000515022 | chr11 |
| ASHG19AP1B101848137V5 | protein_coding | ENST00000375559 | chr13 |
| ASHG19AP1B100026912V5 | protein_coding | ENST00000377311 | chr9 |
| ASHG19AP1B100890801V5 | protein_coding | ENST00000268231 | chr16 |
| ASHG19AP1B136937830V5 | protein_coding | ENST00000373030 | chr20 |
| ASHG19LNC1A109148624V5 | lncRNA | ENST00000593740 | chr19 |
| ASHG19LNC1A100854911V5 | lncRNA | ENST00000584660 | chr17 |
| ASHG19LNC1A102103098V5 | lncRNA | MICT00000132442 | chr16 |
| ASHGV40040540V5 | lncRNA | T285782 | chr5 |
| ASHG19LNC1A100084701V5 | lncRNA | ENST00000520882 | chr5 |
| ASHG19LNC1A100048539V5 | lncRNA | ENST00000606533 | chr1 |
| ASHG19AP1B141920576V5 | protein_coding | ENST00000368704 | chr1 |
| ASHG19LNC1A110630287V5 | lncRNA | ENST00000424174 | chr3 |
| ASHG19LNC1A100015590V5 | lncRNA | ENST00000511650 | chr5 |
| ASHG19LNC1A106045150V5 | lncRNA | ENST00000594398 | chr16 |
| ASHGV40006178V5 | lncRNA | TCONS_00018332 | chr10 |
| ASHG19AP1B113236502V5 | protein_coding | ENST00000520347 | chr8 |
| ASHG19LNC1A102429018V5 | lncRNA | ENST00000593861 | chr2 |
| ASHG19LNC1A100092968V5 | lncRNA | ENST00000605846 | chr1 |
| ASHGV40001413V5 | lncRNA | NR_026562 | chr20 |
| ASHG19LNC1A106399525V5 | lncRNA | ENST00000635395 | chr1 |
| ASHG19LNC1A100063734V5 | lncRNA | ENST00000413269 | chr9 |
| ASHGV40010017V5 | lncRNA | ENST00000512916 | chr12 |
| ASHG19LNC1A100076220V5 | lncRNA | ENST00000459855 | chr3 |
| ASHG19AP1B126722368V5 | protein_coding | ENST00000409299 | chr20 |
| ASHG19LNC1A100052346V5 | lncRNA | ENST00000549659 | chr20 |
| ASHG19LNC1A104465952V5 | lncRNA | ENST00000440490 | chr10 |
| ASHGV40059791V5 | lncRNA | TCONS_00027288 | chr19 |
| ASHG19AP1B116753425V5 | protein_coding | ENST00000553380 | chr9 |
| ASHGV40053220V5 | lncRNA | TCONS_00016098 | chr9 |
| ASHG19AP1B139781923V5 | protein_coding | ENST00000462014 | chr3 |
| ASHG19LNC1A100020163V5 | lncRNA | ENST00000573982 | chr16 |
| ASHG19LNC1A104893279V5 | lncRNA | ENST00000456210 | chr3 |
| ASHG19AP1B124346093V5 | protein_coding | ENST00000343805 | chr2 |
| ASHG19AP1B100227479V5 | protein_coding | ENST00000378453 | chr1 |
| ASHGV40057731V5 | lncRNA | ENST00000500447 | chr11 |
| ASHG19LNC1A107625614V5 | lncRNA | ENST00000557467 | chr14 |
| ASHG19LNC1A101637126V5 | lncRNA | ENST00000421758 | chr13 |
| ASHG19LNC1A110416853V5 | lncRNA | ENST00000497086 | chr1 |
| ASHG19LNC1A100080479V5 | lncRNA | ENST00000510274 | chr5 |
| ASHG19AP1B141555700V5 | protein_coding | ENST00000329421 | chr1 |
| ASHGV40026229V5 | lncRNA | T183354 | chr2 |
| ASHG19LNC1A100076699V5 | lncRNA | ENST00000563605 | chr16 |
| ASHG19AP1B121660446V5 | protein_coding | ENST00000396134 | chr16 |
| ASHG19LNC1A100032732V5 | lncRNA | ENST00000648543 | chr5 |
| ERCC-00097_63 |  |  |  |
| ASHGV40050778V5 | lncRNA | T345441 | chr8 |
| ASHG19LNC1A101637125V5 | lncRNA | ENST00000433070 | chr13 |
| ASHGV40059259V5 | lncRNA | TCONS_00001745 | chr1 |
| ASHG19AP1B100102680V5 | protein_coding | ENST00000327259 | chr11 |
| ASHG19AP1B140228294V5 | protein_coding | ENST00000393825 | chr12 |
| ASHG19LNC1A102825717V5 | lncRNA | ENST00000628002 | chr2 |
| ASHG19AP1B127764973V5 | protein_coding | ENST00000335877 | chr20 |
| ASHG19LNC1A109221690V5 | lncRNA | ENST00000583612 | chr18 |
| ASHG19LNC1A108946762V5 | lncRNA | NR_135769 | chr2 |
| ASHG19LNC1A103685577V5 | lncRNA | ENCT00000072812 | chr11 |
| ASHG19LNC1A100032333V5 | lncRNA | ENST00000566954 | chr9 |
| ASHG19LNC1A100076703V5 | lncRNA | ENST00000566420 | chr6 |
| ASHG19AP1B105392872V5 | protein_coding | ENST00000268661 | chr16 |
| ASHG19AP1B108131779V5 | protein_coding | ENST00000339465 | chr15 |
| ASHG19AP1B109420977V5 | protein_coding | ENCT00000001323 | chr1 |
| ASHG19AP1B102211524V5 | protein_coding | ENST00000296046 | chr3 |
| ASHGV40059449V5 | lncRNA | TCONS_00012022 | chr6 |
| ASHGV40001229V5 | lncRNA | ENST00000457369 | chr2 |
| ASHG19AP1B100262006V5 | protein_coding | ENST00000578689 | chr17 |
| ASHG19AP1B100226035V5 | protein_coding | ENST00000302165 | chr19 |
| ASHG19AP1B116099560V5 | protein_coding | ENST00000339098 | chr2 |
| ASHG19AP1B119162740V5 | protein_coding | ENST00000278572 | chr11 |
| ASHG19LNC1A109158522V5 | lncRNA | ENST00000420876 | chr1 |
| ASHG19LNC1A100454332V5 | lncRNA | ENST00000647756 | chr13 |
| ASHGV40001197V5 | lncRNA | ENST00000455866 | chr7 |
| ASHG19LNC1A100073073V5 | lncRNA | ENCT00000088265 | chr12 |
| ASHG19LNC1A102092106V5 | lncRNA | ENST00000418273 | chr10 |
| ASHG19LNC1A100011085V5 | lncRNA | ENST00000411824 | chr20 |
| ASHG19LNC1A111689598V5 | lncRNA | ENST00000598789 | chr19 |
| ASHG19LNC1A107535210V5 | lncRNA | ENST00000624614 | chr8 |
| ASSPINKEIN100003643 |  |  |  |
| ASHG19AP1B104673574V5 | protein_coding | ENST00000326474 | chr3 |
| ASHG19LNC1A100093030V5 | lncRNA | ENST00000619086 | chr12 |
| ASHG19AP1B115327726V5 | protein_coding | ENST00000221399 | chr19 |
| ASHGV40051007V5 | lncRNA | ENST00000521625 | chr8 |
| ASHGV40052827V5 | lncRNA | T358157 | chr9 |
| ASHGV40033809V5 | lncRNA | ENST00000450750 | chr22 |
| ASHG19LNC1A104002307V5 | lncRNA | ENST00000444958 | chr4 |
| ASHG19AP1B118570399V5 | protein_coding | ENST00000347869 | chr3 |
| ASHG19AP1B102926930V5 | protein_coding | ENST00000621536 | chr5 |
| ASHGV40040841V5 | lncRNA | ENST00000508452 | chr5 |
| ASHG19LNC1A100048727V5 | lncRNA | NR_145459 | chr11 |
| ASHG19AP1B100010099V5 | protein_coding | FTMT24000007118 | chr10 |
| ASHGV40016060V5 | lncRNA | T118794 | chr15 |
| ASHG19AP1B100143862V5 | protein_coding | ENST00000379375 | chr6 |
| ASHG19AP1B127343616V5 | protein_coding | ENST00000614342 | chr14 |
| ASHG19AP1B141086300V5 | protein_coding | ENST00000372098 | chr1 |
| ASHG19AP1B133251296V5 | protein_coding | ENST00000244096 | chrX |
| ASHG19LNC1A107787072V5 | lncRNA | ENST00000544125 | chr12 |
| ASHG19LNC1A100052843V5 | lncRNA | NR_135274 | chr14 |
| ASHG19AP1B116251415V5 | protein_coding | MICT00000233834 | chr22 |
| ASHGV40028330V5 | lncRNA | ENST00000418218 | chr2 |
| ASHG19LNC1A108788791V5 | lncRNA | ENST00000451451 | chr22 |
| ASHG19AP1B100207377V5 | protein_coding | ENST00000309880 | chr11 |
| ASHG19LNC1A109555842V5 | lncRNA | ENST00000648779 | chr2 |
| ASHG19LNC1A100034530V5 | lncRNA | ENST00000470729 | chr2 |
| ASHGV40030424V5 | lncRNA | ENST00000414676 | chr20 |
| ASHGV40034868V5 | lncRNA | TCONS_00006652 | chr3 |
| ASHG19AP1B105033128V5 | protein_coding | ENST00000391910 | chr19 |
| ASHG19LNC1A109552992V5 | lncRNA | ENST00000412196 | chr1 |
| ASHG19LNC1A100072148V5 | lncRNA | ENST00000435157 | chr9 |
| ASHG19AP1B102268874V5 | protein_coding | ENST00000297814 | chr9 |
| ASHG19LNC1A100004110V5 | lncRNA | BIG-lncRNA-582.1 | chr5 |
| ASHG19LNC1A100057076V5 | lncRNA | FTMT26800005115 | chr17 |
| ASHG19LNC1A110290204V5 | lncRNA | ENST00000649712 | chr17 |
| ASHGV40029603V5 | lncRNA | ENST00000427278 | chr2 |
| ASHG19LNC1ABL100000566V5 | lncRNA | compmerge.1904.pooled.chr12 | chr12 |
| ASHG19AP1B108733567V5 | protein_coding | ENST00000253401 | chrX |
| ASHG19LNC1A100887318V5 | lncRNA | ENST00000542112 | chr11 |
| ASHG19AP1B106402435V5 | protein_coding | ENST00000359623 | chr7 |
| ASHG19LNC1A100089080V5 | lncRNA | ENST00000611714 | chr12 |
| ASHG19LNC1A110321113V5 | lncRNA | ENST00000477931 | chr20 |
| ASSPINKEIN100008173 |  |  |  |
| ASHG19AP1B100077547V5 | protein_coding | ENST00000372555 | chr20 |
| ASHG19AP1B135988649V5 | protein_coding | ENST00000397133 | chr17 |
| ASHG19LNC1A100093161V5 | lncRNA | FTMT23800004174 | chr10 |
| ASHG19LNC1A100061364V5 | lncRNA | ENCT00000034967 | chr1 |
| ASHG19LNC1A110168759V5 | lncRNA | NR_147060 | chr10 |
| ASHG19AP1B100095122V5 | protein_coding | ENCT00000297820 | chr3 |
| ASHG19LNC1A108801755V5 | lncRNA | ENST00000596312 | chr4 |
| ASHG19LNC1A100044793V5 | lncRNA | ENST00000649302 | chr2 |
| ASHG19LNC1A112100487V5 | lncRNA | ENST00000416220 | chr7 |
| ASHG19LNC1A100835893V5 | lncRNA | ENST00000447784 | chr13 |
| ASHG19AP1B127249610V5 | protein_coding | ENST00000361361 | chr1 |
| ASHG19LNC1A107590865V5 | lncRNA | ENST00000429932 | chrX |
| ASHG19AP1B102078045V5 | protein_coding | ENST00000369878 | chr10 |
| ASHG19LNC1A100076086V5 | lncRNA | ENST00000436582 | chr2 |
| ASHG19AP1B100171364V5 | protein_coding | ENCT00000142483 | chr15 |
| ASHG19LNC1A106002067V5 | lncRNA | ENST00000468141 | chr2 |
| ASHG19AP1B118160980V5 | protein_coding | ENST00000294119 | chr11 |
| ASHG19LNC1A100012534V5 | lncRNA | HBMT00000071592 | chr1 |
| ASHG19AP1B100044033V5 | protein_coding | ENST00000293261 | chr19 |
| ASHG19LNC1A101254421V5 | lncRNA | ENST00000634569 | chr1 |
| ASHG19AP1B101393687V5 | protein_coding | ENST00000450269 | chr1 |
| ASHG19LNC1A106434347V5 | lncRNA | ENST00000558888 | chr15 |
| ASHG19LNC1A112379221V5 | lncRNA | ENST00000578001 | chr17 |
| ASHG19AP1B132472269V5 | protein_coding | ENST00000367649 | chr1 |
| ASHG19LNC1A100057683V5 | lncRNA | ENST00000485482 | chr22 |
| ASHGV40051914V5 | lncRNA | T361342 | chr9 |
| ASHG19LNC1A100075651V5 | lncRNA | ENST00000437080 | chr1 |
| ASHG19AP1B104650775V5 | protein_coding | ENST00000308064 | chr11 |
| ASHG19LNC1A110644885V5 | lncRNA | ENST00000576021 | chr17 |
| ASHG19LNC1A101660177V5 | lncRNA | ENST00000625703 | chr3 |
| ASHG19AP1B124077767V5 | protein_coding | ENST00000202677 | chr20 |
| ASHG19AP1B117393755V5 | protein_coding | ENCT00000161152 | chr16 |
| ASHGV40004388V5 | lncRNA | NR_110246 | chr2 |
| ASHG19AP1B120812793V5 | protein_coding | FTMT28800000290 | chr22 |
| ASHG19AP1B110829606V5 | protein_coding | ENST00000396667 | chr7 |
| ASHG19LNC1A100028094V5 | lncRNA | ENST00000543604 | chr12 |
| ASHG19LNC1A106532476V5 | lncRNA | ENST00000519409 | chr8 |
| ASHG19AP1B100140590V5 | protein_coding | ENST00000311923 | chr8 |
| ASHG19AP1B104000725V5 | protein_coding | ENST00000591228 | chr17 |
| ASHGV40023539V5 | lncRNA | ENST00000563172 | chr18 |
| ASHG19LNC1A102866939V5 | lncRNA | ENST00000549551 | chr12 |
| ASHG19LNC1A106013140V5 | lncRNA | NR_146637 | chr3 |
| ASHGV40048341V5 | lncRNA | ENST00000435981 | chr7 |
| ASHG19AP1B133038450V5 | protein_coding | ENST00000354321 | chr17 |
| ASHGV40021624V5 | lncRNA | NR_111970 | chr17 |
| ASHG19LNC1A102085612V5 | lncRNA | ENST00000570314 | chr17 |
| ASHG19LNC1A102428297V5 | lncRNA | ENST00000443587 | chr13 |
| ASHG19AP1B101019195V5 | protein_coding | ENST00000398246 | chr8 |
| ASHG19LNC1A100088080V5 | lncRNA | ENST00000440798 | chr20 |
| ASHG19LNC1A100482211V5 | lncRNA | ENST00000513626 | chr5 |
| ASHG19AP1B100197615V5 | protein_coding | ENST00000436693 | chr4 |
| ASHG19LNC1A100072825V5 | lncRNA | ENST00000614509 | chr15 |
| ASHG19LNC1A106475763V5 | lncRNA | ENST00000464290 | chr9 |
| ASHG19LNC1A105245011V5 | lncRNA | ENST00000547040 | chr12 |
| ASHG19LNC1A100012092V5 | lncRNA | ENST00000609511 | chr4 |
| ASHG19AP1B143197067V5 | protein_coding | ENST00000411763 | chr3 |
| ASHG19LNC1A102812538V5 | lncRNA | ENST00000638047 | chr2 |
| ASHG19LNC1ABL100000870V5 | lncRNA | compmerge.4590.pooled.chr7 | chr7 |
| ASHG19AP1B111702966V5 | protein_coding | MICT00000178407 | chr19 |
| ASHG19LNC1A100084341V5 | lncRNA | ENST00000594488 | chr13 |
| ASHGV40019012V5 | lncRNA | T131710 | chr16 |
| ASHG19AP1B108818018V5 | protein_coding | ENST00000308580 | chr15 |
| ASHG19LNC1A106039798V5 | lncRNA | ENST00000566411 | chr16 |
| ASHG19LNC1ABL100000681V5 | lncRNA | compmerge.2790.pooled.chr7 | chr7 |
| ASHG19AP1B107974207V5 | protein_coding | ENST00000409607 | chr7 |
| ASHGV40024194V5 | lncRNA | TCONS_00027289 | chr19 |
| ASHG19AP1B102930566V5 | protein_coding | ENST00000424196 | chr3 |
| ASHG19LNC1A100060784V5 | lncRNA | ENST00000602760 | chr2 |
| ASHG19AP1B124609586V5 | protein_coding | ENST00000366630 | chr1 |
| ASHG19AP1B101292487V5 | protein_coding | ENST00000619644 | chr22 |
| ASHGV40037100V5 | lncRNA | ENST00000489312 | chr4 |
| ASHG19LNC1A109427515V5 | lncRNA | ENST00000472613 | chr1 |
| ASHG19AP1B100785967V5 | protein_coding | ENST00000258499 | chr12 |
| ASHG19AP1B142926223V5 | protein_coding | ENST00000330387 | chr7 |
| ASHG19LNC1A108832336V5 | lncRNA | ENST00000638931 | chr1 |
| ASHG19AP1B103461095V5 | protein_coding | ENST00000309680 | chr12 |
| ASHGV40029368V5 | lncRNA | ENST00000412690 | chr2 |
| ASHG19LNC1A104876954V5 | lncRNA | ENCT00000262354 | chr20 |
| ASHG19LNC1A109294555V5 | lncRNA | ENST00000550404 | chr12 |
| ASHG19LNC1A100028099V5 | lncRNA | ENST00000549104 | chr10 |
| ASHGV40051026V5 | lncRNA | ENST00000520820 | chr8 |
| ASHG19LNC1ABL100000390V5 | lncRNA | HSALNT0289256 | chr12 |
| ASHG19LNC1A108131956V5 | lncRNA | ENST00000579096 | chr17 |
| ASHG19AP1B100219166V5 | protein_coding | MICT00000217507 | chr20 |
| ASHG19AP1B134807541V5 | protein_coding | ENST00000617752 | chr1 |
| ASHG19AP1B124487458V5 | protein_coding | ENST00000354489 | chr6 |
| ASHG19AP1B116871577V5 | protein_coding | ENST00000355528 | chr17 |
| ASHGV40050040V5 | lncRNA | T352642 | chr8 |
| ASHG19LNC1A100043936V5 | lncRNA | ENST00000442435 | chr1 |
| ASHG19LNC1A100024725V5 | lncRNA | FTMT24600000467 | chr12 |
| ASHG19LNC1A109170827V5 | lncRNA | ENST00000453417 | chr6 |
| ASHG19AP1B127674344V5 | protein_coding | ENST00000450142 | chr12 |
| ASHG19AP1B112128398V5 | protein_coding | ENST00000243457 | chr17 |
| ASHG19LNC1ABL100000341V5 | lncRNA | HSALNT0289202 | chr2 |
| ASHG19AP1B114603136V5 | protein_coding | ENST00000277526 | chr9 |
| ASHG19LNC1A107597508V5 | lncRNA | ENST00000380121 | chr13 |
| ASHG19LNC1A106435265V5 | lncRNA | ENST00000563993 | chr16 |
| ASHG19LNC1A100025009V5 | lncRNA | FTMT20400001693 | chr1 |
| ASHGV40027552V5 | lncRNA | ENST00000441356 | chr2 |
| ASHG19LNC1A100048535V5 | lncRNA | ENST00000605056 | chr2 |
| ASHG19AP1B100132859V5 | protein_coding | ENST00000620492 | chr16 |
| ASHG19AP1B140078097V5 | protein_coding | ENST00000372358 | chr9 |
| ASHG19LNC1A100032095V5 | lncRNA | ENST00000517300 | chr8 |
| ASHG19AP1B110408797V5 | protein_coding | ENST00000333503 | chr16 |
| ASHG19LNC1A100002945V5 | lncRNA | ENST00000446262 | chr19 |
| ASHG19LNC1A113684843V5 | lncRNA | ENST00000553351 | chr14 |
| ASHG19LNC1A104055872V5 | lncRNA | ENST00000548665 | chr14 |
| ASHGV40004359V5 | lncRNA | NR_110110 | chr12 |
