# Supplementary material for: Understanding Gene Expression and Transcriptome Profiling of COVID-19: An Initiative Towards the Mapping of Protective Immunity Genes Against SARS-CoV-2 Infection
Source: Front Immunol. 2021 Dec 15;12:724936. doi: 10.3389/fimmu.2021.724936 (PMC8714830; doi:10.3389/fimmu.2021.724936)
Supplement: Supplementary file 4 [file Table_4.docx]

**Table S4.** Protein coding gene and their NCBI accession number, gene name.

| **Sl. No.** | **Accession no** | **Gene name** |
| --- | --- | --- |
|  | ENST00000255039 | HAPLN2 |
|  | ENST00000320838 | KLK8 |
|  | NM_001318905 | GLUD1 |
|  | ENST00000342782 | DUSP9 |
|  | ENST00000262746 | PRDX1 |
|  | ENST00000369076 | ATG5 |
|  | ENST00000602198 | TEX101 |
|  | ENST00000536005 | BEAN1 |
|  | ENST00000337665 | ARHGEF1 |
|  | ENST00000515022 | TRIM6 |
|  | ENST00000375559 | F10 |
|  | ENST00000377311 | TMEM252 |
|  | ENST00000268231 | SEPTIN12 |
|  | ENST00000373030 | IFT52 |
|  | ENST00000368704 | S100A16 |
|  | ENST00000520347 | ZNF706 |
|  | ENST00000553380 | ZFP37 |
|  | ENST00000462014 |  |
|  | ENST00000343805 | SP140 |
|  | ENST00000378453 | HES5 |
|  | ENST00000329421 | MARCKSL1 |
|  | ENST00000396134 | UMOD |
|  | ENST00000327259 | TMEM151A |
|  | ENST00000393825 | NDUFA4L2 |
|  | ENST00000335877 | PSMF1 |
|  | ENST00000268661 | RPL3L |
|  | ENST00000339465 | SAXO2 |
|  | ENCT00000001323 |  |
|  | ENST00000296046 | CPA3 |
|  | ENST00000578689 | GJD3 |
|  | ENST00000302165 | IRF2BP1 |
|  | ENST00000339098 | CERKL |
|  | ENST00000278572 | RPS3 |
|  | ENST00000326474 | C3ORF80 |
|  | ENST00000221399 | TULP2 |
|  | ENST00000347869 | RBM5 |
|  | ENST00000621536 | FGF1 |
|  | ENST00000379375 | EDN1 |
|  | ENST00000614342 | SALL2 |
|  | ENST00000372098 | MUTYH |
|  | ENST00000244096 | MAGEA10 |
|  | ENST00000309880 | TIGD3 |
|  | ENST00000391910 | STRN4 |
|  | ENST00000297814 | KIF27 |
|  | ENST00000253401 | ARHGEF9 |
|  | ENST00000359623 | LRRC61 |
|  | ENST00000372555 | TNNC2 |
|  | ENST00000397133 | EMC6 |
|  | ENST00000361361 | FAM189B |
|  | ENST00000369878 | CNNM2 |
|  | ENST00000294119 | UBXN1 |
|  | ENST00000293261 | TMEM143 |
|  | ENST00000450269 | BTBD19 |
|  | ENST00000367649 | RASAL2 |
|  | ENST00000308064 | CHST1 |
|  | ENST00000202677 | RALGAPA2 |
|  | ENST00000396667 | TMEM106B |
|  | ENST00000311923 | MOS |
|  | ENST00000591228 | PPY |
|  | ENST00000354321 | DUS1L |
|  | ENST00000398246 | LONRF1 |
|  | ENST00000436693 | TLR6 |
|  | ENST00000411763 | AP2M1 |
|  | ENST00000308580 | TMOD3 |
|  | ENST00000409607 | LLCFC1 |
|  | ENST00000424196 | EIF4G1 |
|  | ENST00000366630 | SIPA1L2 |
|  | ENST00000619644 | SMTN |
|  | ENST00000258499 | USP44 |
|  | ENST00000330387 | CREB3L2 |
|  | ENST00000309680 | KRT2 |
|  | ENST00000617752 | OR2C3 |
|  | ENST00000354489 | MAK |
|  | ENST00000355528 | TBCD |
|  | ENST00000450142 | PPP1R12A |
|  | ENST00000243457 | KCNJ2 |
|  | ENST00000277526 | LCN9 |
|  | ENST00000620492 | TMEM114 |
|  | ENST00000372358 | EXOSC2 |
|  | ENST00000333503 | PGP |
