# Supplementary material for: Understanding Gene Expression and Transcriptome Profiling of COVID-19: An Initiative Towards the Mapping of Protective Immunity Genes Against SARS-CoV-2 Infection
Source: Front Immunol. 2021 Dec 15;12:724936. doi: 10.3389/fimmu.2021.724936 (PMC8714830; doi:10.3389/fimmu.2021.724936)
Supplement: Supplementary file 5 [file Table_5.docx]

**Table S5.** Long non-coding RNA gene and their NCBI accession number, gene name.

| **Sl. No.** | **Accession no** | **Gene name** |
| --- | --- | --- |
|  | NR_110947 | LOC101929613 |
|  | NR_148363 | LOC105370401 |
|  | FTMT21000006986 | - |
|  | ENCT00000222744 | - |
|  | ENST00000608176 | - |
|  | T097905 | - |
|  | ENST00000649453 | - |
|  | ENST00000412295 | - |
|  | ENST00000562514 | - |
|  | ENST00000419183 | - |
|  | ENST00000555460 | - |
|  | ENST00000509458 | - |
|  | ENST00000619099 |  |
|  | ENST00000593740 | - |
|  | ENST00000584660 | - |
|  | MICT00000132442 | - |
|  | T285782 | - |
|  | ENST00000520882 | - |
|  | ENST00000606533 | - |
|  | ENST00000424174 | - |
|  | ENST00000511650 | - |
|  | ENST00000594398 | - |
|  | TCONS_00018332 | - |
|  | ENST00000593861 | - |
|  | ENST00000605846 | - |
|  | NR_026562 | RAB5IF |
|  | ENST00000635395 | - |
|  | ENST00000413269 | TMEM252-DT |
|  | ENST00000512916 | HOXC13-AS |
|  | ENST00000459855 | - |
|  | ENST00000549659 | - |
|  | ENST00000440490 | - |
|  | TCONS_00027288 | - |
|  | TCONS_00016098 | - |
|  | ENST00000573982 | - |
|  | ENST00000456210 | SEMA3B |
|  | ENST00000500447 | MIR210HG |
|  | ENST00000557467 | - |
|  | ENST00000421758 | DLEU2 |
|  | ENST00000497086 | - |
|  | ENST00000510274 | MEF2C-AS2 |
|  | T183354 | - |
|  | ENST00000563605 | - |
|  | ENST00000648543 | - |
|  | T345441 | - |
|  | ENST00000433070 | - |
|  | TCONS_00001745 | - |
|  | ENST00000628002 | - |
|  | ENST00000583612 | - |
|  | NR_135769 | HTRA2 |
|  | ENCT00000072812 |  |
|  | ENST00000566954 | - |
|  | ENST00000566420 | - |
|  | TCONS_00012022 | - |
|  | ENST00000457369 | - |
|  | ENST00000420876 | - |
|  | ENST00000647756 | - |
|  | ENST00000455866 | - |
|  | ENCT00000088265 | - |
|  | ENST00000418273 | - |
|  | ENST00000411824 | - |
|  | ENST00000598789 | - |
|  | ENST00000624614 | - |
|  | ENST00000619086 | - |
|  | ENST00000521625 | - |
|  | T358157 | - |
|  | ENST00000450750 | LINC01639 |
|  | ENST00000444958 | - |
|  | ENST00000508452 | SLC25A48-AS1 |
|  | NR_145459 | TALAM1 |
|  | T118794 | - |
|  | ENST00000544125 | - |
|  | NR_135274 | LOC105370619 |
|  | ENST00000418218 | UICLM |
|  | ENST00000451451 | - |
|  | ENST00000648779 | - |
|  | ENST00000470729 | ANKRD30BL |
|  | ENST00000414676 | NRSN2-AS1 |
|  | TCONS_00006652 | - |
|  | ENST00000412196 | - |
|  | ENST00000435157 | - |
|  | BIG-lncRNA-582.1 | - |
|  | FTMT26800005115 | - |
|  | ENST00000649712 | SNHG20 |
|  | ENST00000427278 | - |
|  | ENST00000542112 | - |
|  | ENST00000611714 | - |
|  | ENST00000477931 | GNAS |
|  | FTMT23800004174 | - |
|  | ENCT00000034967 | - |
|  | NR_147060 | MEIG1 |
|  | ENST00000596312 | - |
|  | ENST00000649302 | - |
|  | ENST00000416220 | - |
|  | ENST00000447784 | - |
|  | ENST00000436582 | - |
|  | ENST00000468141 | - |
|  | HBMT00000071592 | - |
|  | ENST00000634569 | - |
|  | ENST00000558888 | - |
|  | ENST00000578001 | - |
|  | ENST00000485482 | - |
|  | T361342 | - |
|  | ENST00000437080 | - |
|  | ENST00000576021 | - |
|  | ENST00000625703 | - |
|  | NR_110246 | LINC02612 |
|  | ENST00000543604 | - |
|  | ENST00000519409 | - |
|  | ENST00000563172 | LINC02582 |
|  | ENST00000549551 |  |
|  | NR_146637 | LINC0200 |
|  | ENST00000435981 | LINC01393 |
|  | NR_111970 | CCL4L2 |
|  | ENST00000570314 | - |
|  | ENST00000443587 | - |
|  | ENST00000440798 | -- |
|  | ENST00000513626 | - |
|  | ENST00000614509 | - |
|  | ENST00000464290 | - |
|  | ENST00000547040 | - |
|  | ENST00000609511 | - |
|  | ENST00000638047 | - |
|  | ENST00000594488 | - |
|  | T131710 | - |
|  | ENST00000566411 | - |
|  | TCONS_00027289 | - |
|  | ENST00000602760 | - |
|  | ENST00000489312 | - |
|  | ENST00000472613 | - |
|  | ENST00000638931 | - |
|  | ENST00000412690 | LINC01191 |
|  | ENCT00000262354 | - |
|  | ENST00000550404 | - |
|  | ENST00000549104 | - |
|  | ENST00000520820 | - |
|  | HSALNT0289256 | - |
|  | ENST00000579096 | - |
|  | T352642 | - |
|  | ENST00000442435 | - |
|  | FTMT24600000467 | - |
|  | ENST00000453417 | - |
|  | HSALNT0289202 | - |
|  | ENST00000380121 | N4BP2L2 |
|  | ENST00000563993 | - |
|  | FTMT20400001693 | - |
|  | ENST00000441356 | LINC01920 |
|  | ENST00000605056 | - |
|  | ENST00000517300 | - |
|  | ENST00000446262 | - |
|  | ENST00000553351 | - |
|  | ENST00000548665 | - |
|  | NR_110110 | CASC18 |
